# Supplementary material for: Programmable human histone phosphorylation and gene activation using a CRISPR/Cas9-based chromatin kinase
Source: Nat Commun. 2021 Feb 9;12:896. doi: 10.1038/s41467-021-21188-2 (PMC7873277; doi:10.1038/s41467-021-21188-2)
Supplement: Supplementary file 1 — Supplementary Information [file 41467_2021_21188_MOESM1_ESM.pdf]

SUPPLEMENTAL INFORMATION

Programmable human histone phosphorylation and gene activation using a CRISPR/Cas9-based chromatin kinase

Jing Li[1], Barun Mahata[1], Mario Escobar[2], Jacob Goell[1], Kaiyuan Wang[1], Pranav Khemka[1], Isaac B. Hilton[1, 2*]

[1]Department of Bioengineering, Rice University, Houston, TX, USA

[2]Department of BioSciences, Rice University, Houston, TX, USA

*isaac.hilton@rice.edu

Supplementary Figure 1

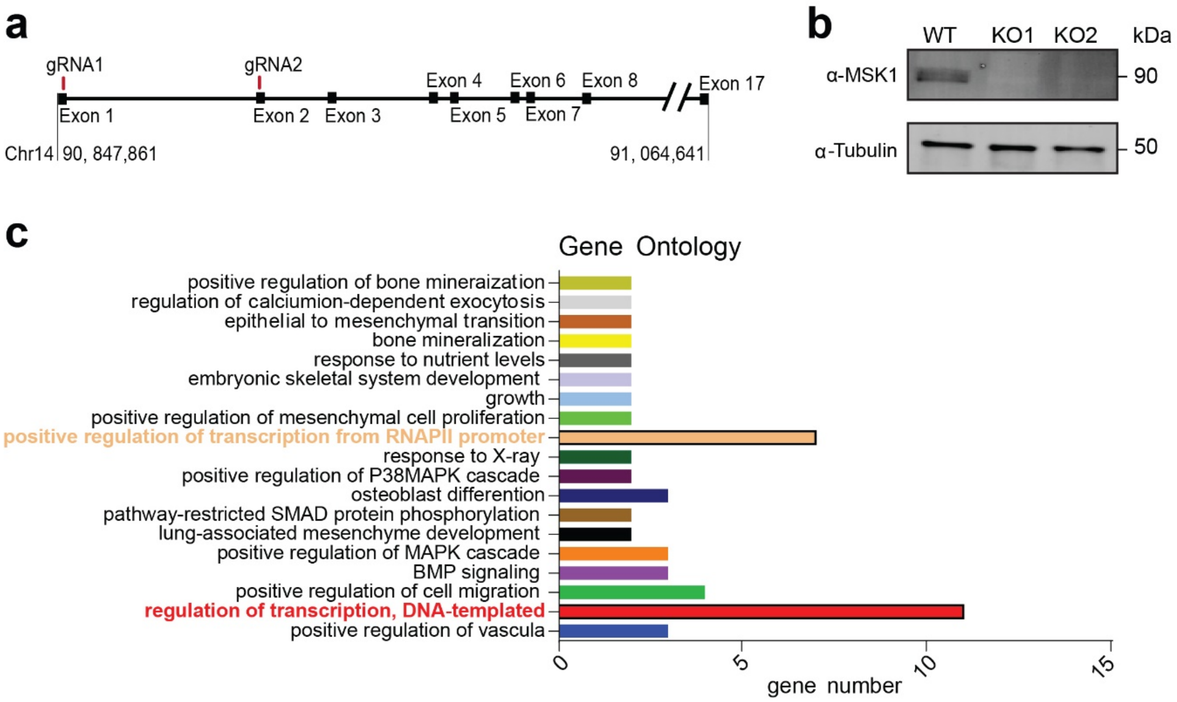

Supplementary Figure 1. Construction and transcriptomic evaluation of MSK1 knockout human cell lines. a. A schematic of the human *MSK1* locus on chromosome 14 is shown. Two guide RNAs (gRNAs) targeting exons 1 or 2 of the *MSK1* gene were designed for CRISPR/Cas9-mediated knockout (KO) of human MSK1. **b.** Western blot analysis of MSK1 protein expression in WT HEK293T cells and two different clonal MSK1 KO HEK293T cell lines (corresponding to gRNA 1 or 2 used for KO). kDa, kilodaltons. Data are representative of 3 independent experiments. **c.** Gene ontology analysis of differentially expressed genes identified from RNA-seq using DAVID Functional Annotation Bioinformatics Microarray Analysis (https://david.ncifcrf.gov/).

Supplementary Figure 2

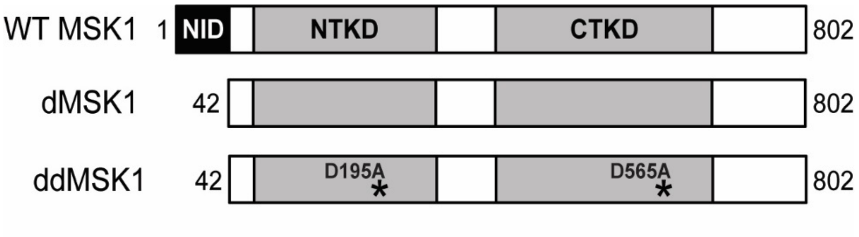

Supplementary Figure 2. Schematic of characterized motifs within the human MSK1 protein. WT human MSK1 contains an N-terminal inhibitory domain (NID), and N and C-terminal kinase domains (NTKD and CTKD, respectively). dMSK1 in this study lacks the NID, and ddMSK1 lacks the NID and harbors two mutations (D195A and D565A) that abolish kinase activity. Numbers correspond to amino acid positions.

Supplementary Figure 3

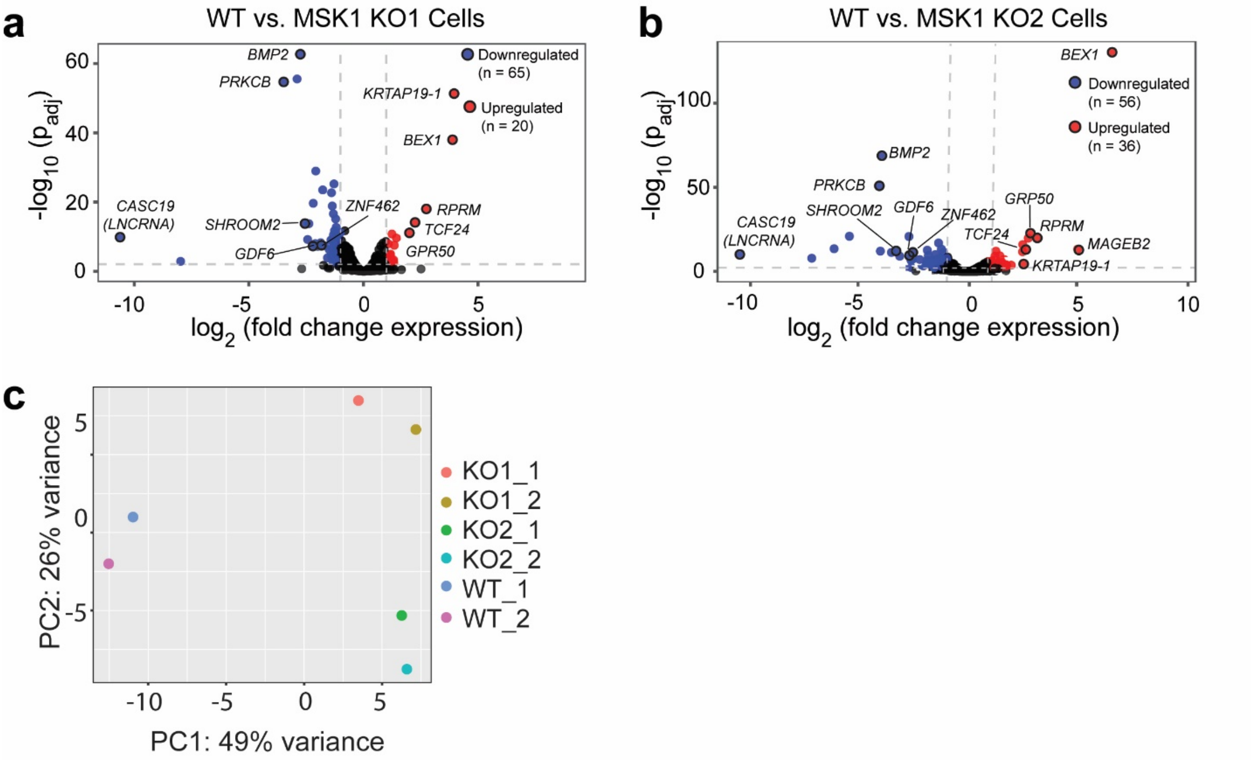

Supplementary Figure 3. MSK1 deletion reproducibly alters the transcriptome of HEK293T cells. a, b. Volcano plots showing differentially expressed genes in different MSK1 knockout cell lines (KO1 and KO2, respectively) relative to WT cells (upregulated genes are depicted as red circles and downregulated genes as blue circles). Data was analyzed using the Wald test and the adjusted *P* value (P_{adj}) was derived using the Benjamini and Hochberg method. **c.** Principal component analysis (PCA) of gene expression between WT and MSK1 KO clones.

Supplementary Figure 4

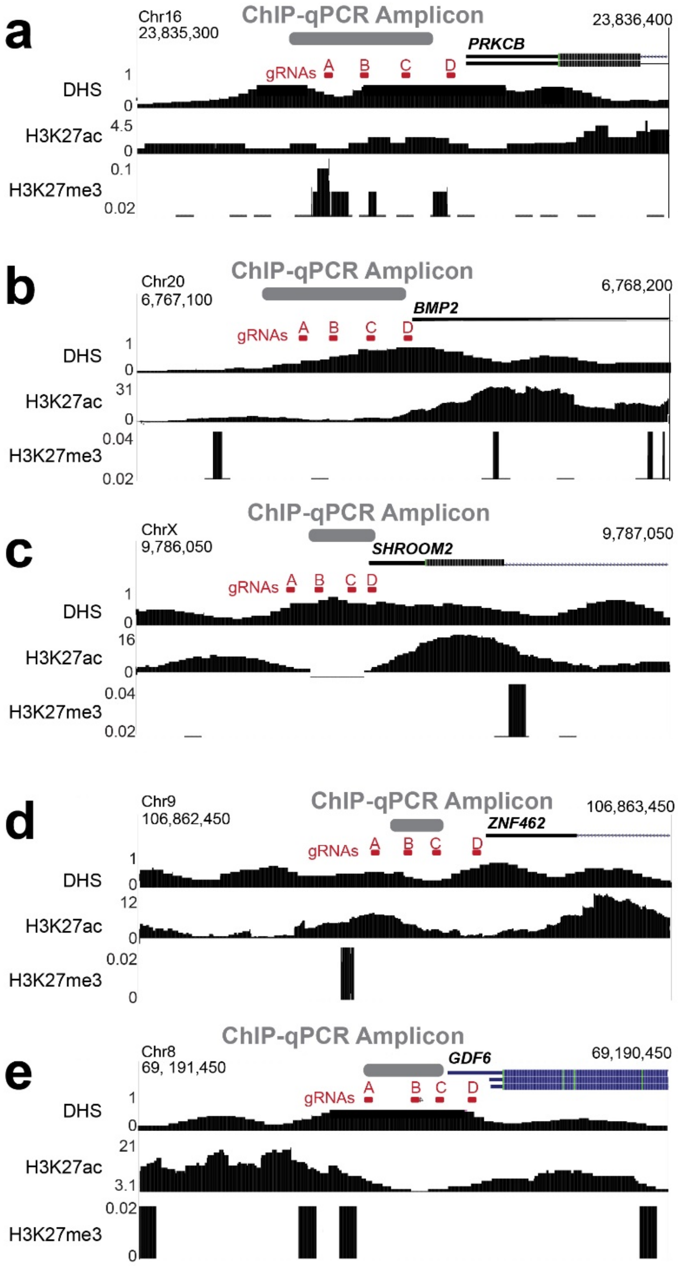

Supplementary Figure 4. Genomic location and chromatin context of gRNAs targeting the top 5 downregulated genes in MSK1 KO cells. a-e. The genomic regions encompassing *PRKCB*, *BMP2*, *SHROOM2*, *GDF6,* and *ZNF462* promoters are shown. Loci are shown along with associated gRNAs (in red) used to target *Streptococcus pyogenes* dCas9 and dCas9-MSK1 fusion proteins. Genomic coordinates are based upon GRCh38/hg38. HEK293T DNase hypersensitivity (DHS; from ENCSR000EJR), HEK293 H3K27ac (from ENCSR000FCH) and HEK293 H3K27me3 (from ChIP-Atlas, DRX013192) levels are also shown. gRNA locations are shown in red. ChIP-qPCR amplicons are shown as grey bars.

Supplementary Figure 5

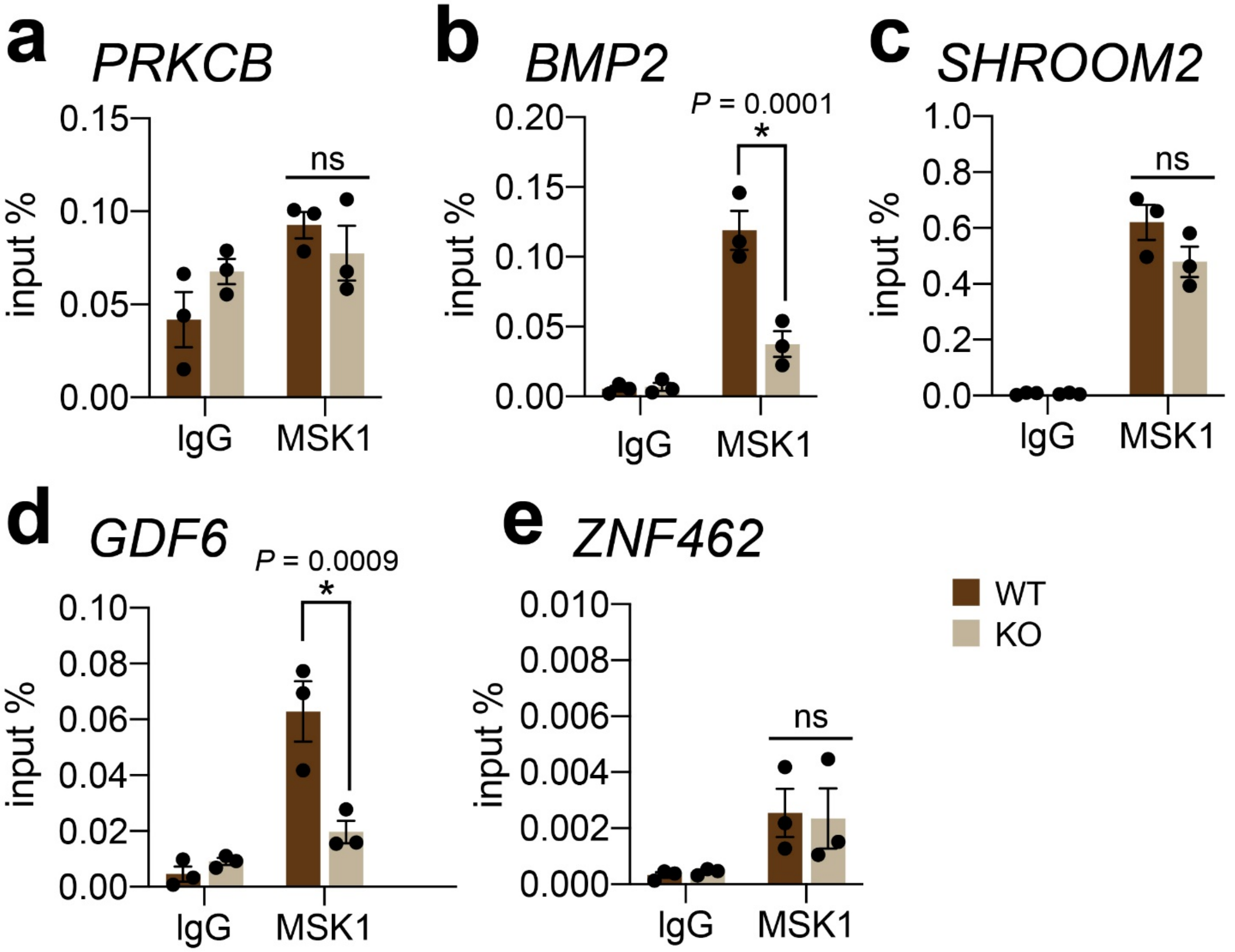

Supplementary Figure 5. MSK1 occupancy at the top 5 downregulated genes in WT and MSK1 KO human cells. a-e. ChIP-qPCR for MSK1 enrichment at the *PRKCB*, *BMP2*, *SHROOM2*, *GDF6*, and *ZNF462* promoters, respectively in WT (brown) or MSK1 KO cells (cream) is shown. 2-sided *t*-test, *$P < 0.05$; n = 3 independent experiments; error bars, s.e.m.; ns, not significant.

Supplementary Figure 6

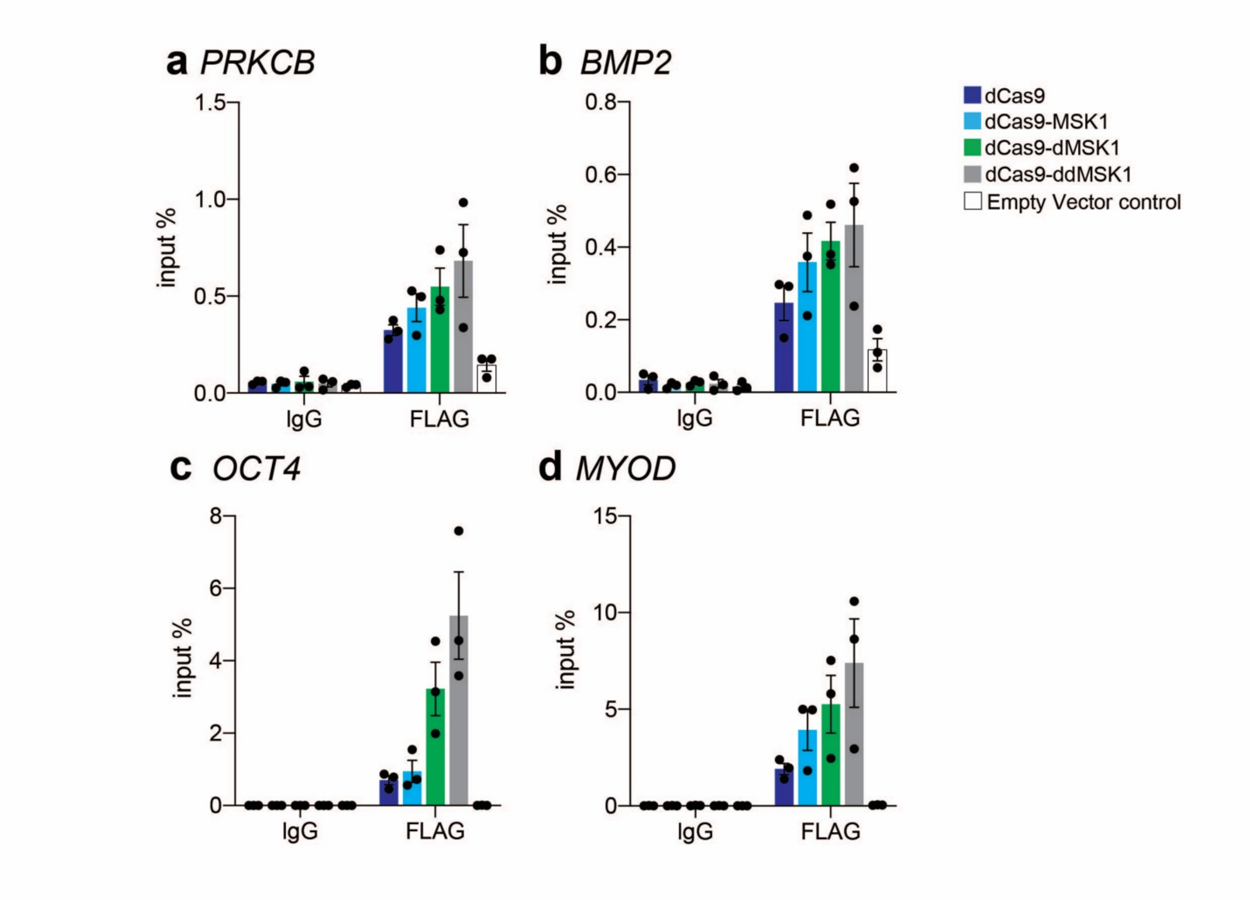

Supplementary Figure 6. dCas9 and dCas9-MSK1 fusion protein variants bind to targeted loci. a-d. ChIP-qPCR for FLAG epitope tag enrichment at the *PRKCB*, *BMP2*, *OCT4* and *MYOD* promoters, respectively is shown. Cells were assayed 72 hours post-transfection with the indicated dCas9-fusion proteins (or dCas9 control). n = 3 independent experiments for all panels; error bars, s.e.m.

Supplementary Figure 7

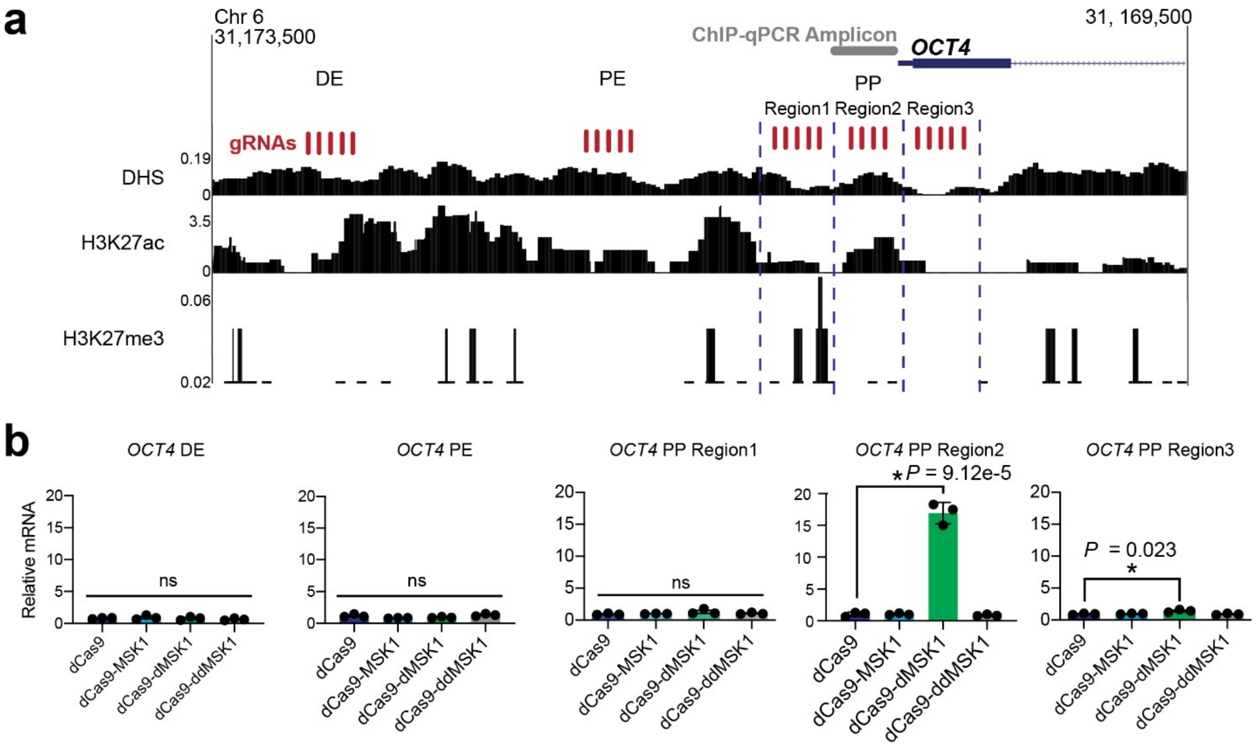

Supplementary Figure 7. dCas9-dMSK1 activates gene expression from the *OCT4* promoter region. a. The genomic region encompassing the distal enhance (DE), proximal enhancer (PE) and proximal promoter (PP) regions governing expression of *OCT4* are shown along with associated gRNAs used in this study. Genomic coordinates are based upon GRCh38/hg38. HEK293T DNase hypersensitivity (DHS; from ENCSR000EJR), HEK293 H3K27ac (from ENCSR000FCH) and HEK293 H3K27me3 (from ChIP-Atlas, DRX013192) levels are also shown and gRNA locations are indicated in red. The amplicon used for ChIP-qPCR is shown as a grey bar. **b.** Relative *OCT4* mRNA was measured by RT-qPCR 72 hours after co-transfection of the indicated dCas9-MSK1 fusions (or dCas9 control) and corresponding gRNAs. 2-sided *t*-test, **P* <0.05; n = 3 independent experiments; error bars, s.e.m.; ns, not significant.

Supplementary Figure 8

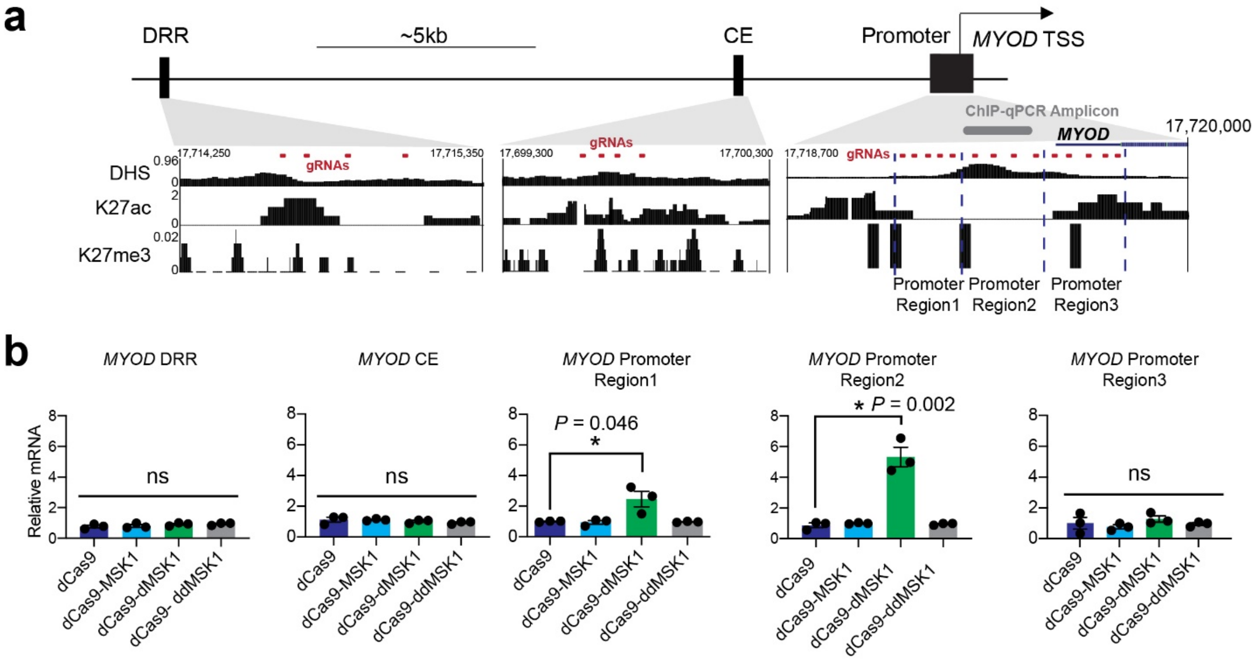

Supplementary Figure 8. dCas9-dMSK1 activates gene expression from the *MYOD* promoter region. a. The genomic region encompassing the distal regulatory region (DRR), core enhancer (CE), and proximal promoter of the *MYOD* gene are shown along with the associated gRNAs used in this study. Genomic coordinates are based upon GRCh38/hg38. HEK293T DNase hypersensitivity (DHS; from ENCSR000EJR), HEK293 H3K27ac (from ENCSR000FCH) and HEK293 H3K27me3 (from ChIP-Atlas, DRX013192) levels are also shown and gRNA locations are indicated in red. The amplicon used for ChIP-qPCR is shown as a grey bar. **b.** Relative *MYOD* mRNA was measured by RT-qPCR 72 hours after co-transfection of indicated MSK1 dCas9 fusions (or dCas9 control) and corresponding gRNAs. 2-sided *t*-test, $^*P < 0.05$; n = 3 independent experiments; error bars, s.e.m.; ns, not significant.

Supplementary Figure 9

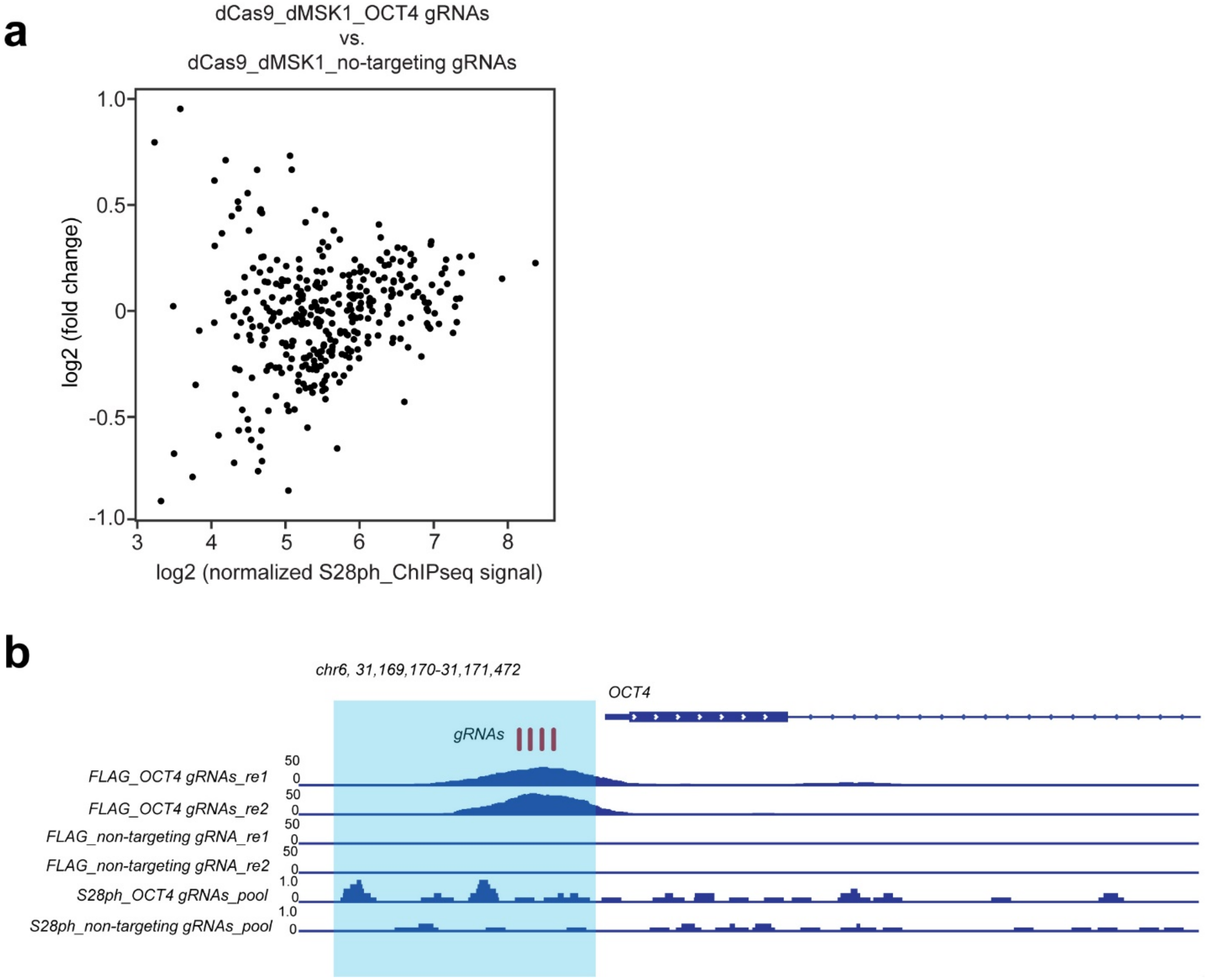

Supplementary Figure 9. ChIP-seq to evaluate the specificity of dCas9-dMSK1-mediated H3S28ph at the *OCT4* promoter. a. DESeq2 analysis of H3S28ph ChIP-seq data from HEK293T cells transiently co-transfected with dCas9-dMSK1 and four *OCT4* promoter-targeting gRNAs compared to HEK293T cells transiently co-transfected with dCas9-dMSK1 and a non-targeting gRNA. **b.** The genomic region encompassing the promoter of the *OCT4* gene is shown along with associated gRNAs. Genomic coordinates are based upon GRCh38/hg38. ChIP-seq signals of FLAG and H3S28ph from HEK293T cells transiently co-transfected with dCas9-dMSK1 and four *OCT4* promoter-targeting gRNAs and HEK293T cells transiently co-transfected with dCas9-dMSK1 and a non-targeting gRNA are shown. gRNA locations are indicated in red.

Supplementary Figure 10

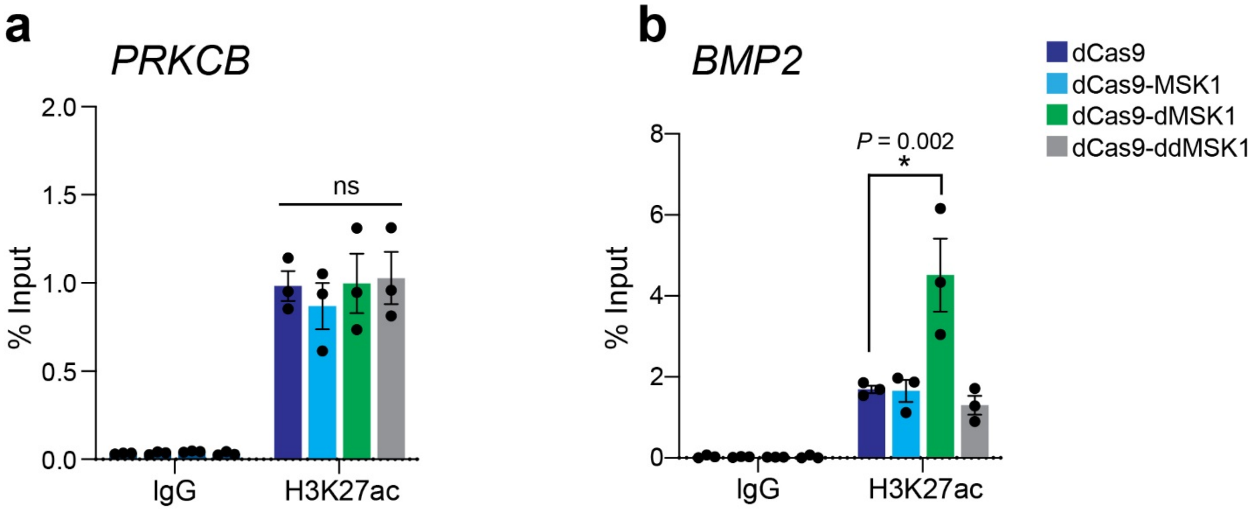

Supplementary Figure 10. dCas9-dMSK1 results in elevated H3K27ac levels at histone phosphorylation-sensitive promoters. a, b. ChIP-qPCR for H3K27ac enrichment at the *PRKCB* and *BMP2* promoters, respectively, 72 hours post-transfection with the indicated dCas9-fusion proteins (or dCas9 control). 2-sided *t*-test, **P* < 0.05; n = 3 independent experiments; error bars, s.e.m.; ns, not significant.

Supplementary Figure 11

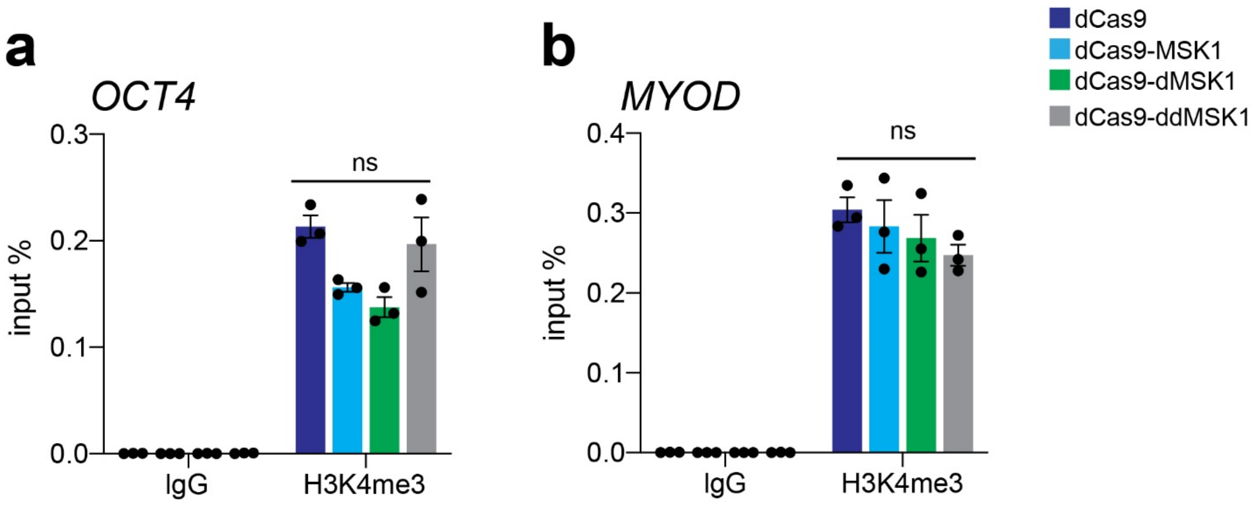

Supplementary Figure 11. dCas9-dMSK1 does not result in increased H3K4me3 levels at tested human promoters. a, b. ChIP-qPCR for H3K4me3 enrichment at the *OCT4* and *MYOD* promoters, respectively, 72 hours post-transfection with the indicated dCas9-fusion proteins (or dCas9 control). One-way ANOVA analysis, n = 3 independent experiments; error bars, s.e.m.; ns, not significant.

Supplementary Figure 12

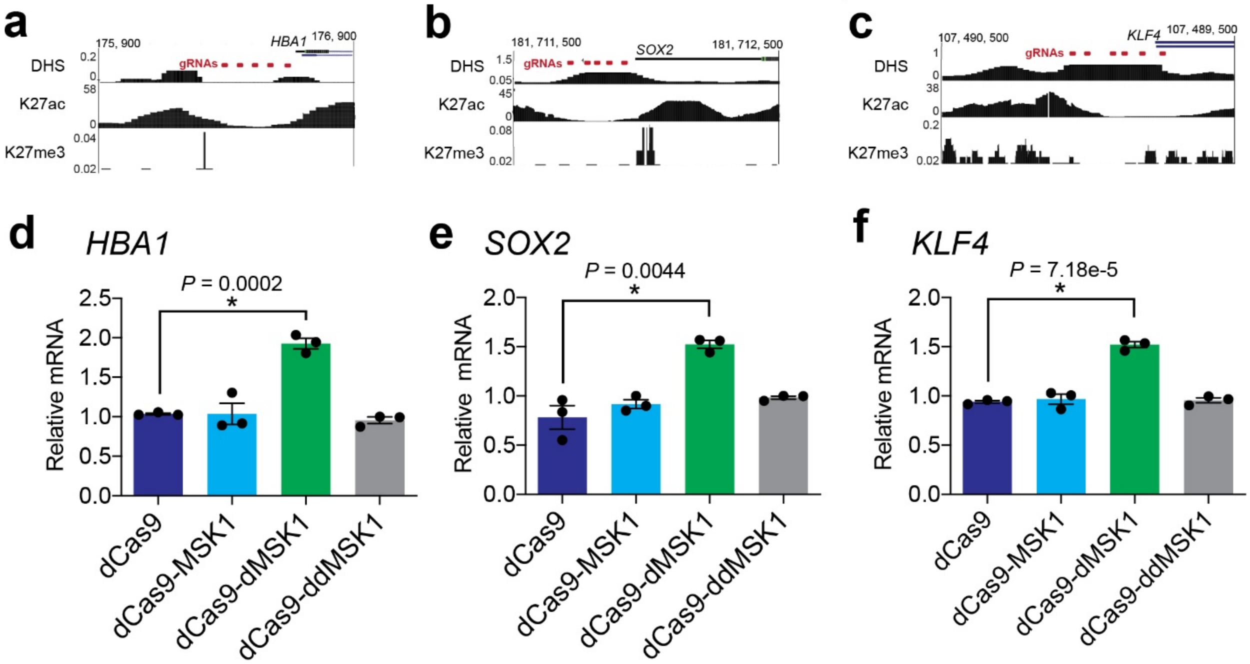

Supplementary Figure 12. dCas9-dMSK1 activates the promoter regions of *HBA1*, *SOX2*, and *KLF4* in HEK293T cells. a-c. The genomic regions encompassing the human *HBA1*, *SOX2,* and *KLF4* promoter regions are shown. Loci are depicted along with gRNAs used in this study. Genomic coordinates are based upon GRCh38/hg38. HEK293T DNase hypersensitivity (DHS; from ENCSR000EJR), HEK293 H3K27ac (from ENCSR000FCH) and HEK293 H3K27me3 (from ChIP-Atlas, DRX013192) levels are also shown and gRNA locations are indicated in red. **d-f**. RT-qPCR for *HBA1*, *SOX2*, and *KLF4* mRNA levels 72 hours post-transfection of dCas9, dCas9-MSK1, dCas9-dMSK1, or dCas9-ddMSK1 and corresponding gRNAs. 2-sided *t*-test, $*P < 0.05$; n = 3 independent experiments for all panels; error bars, s.e.m.; ns, not significant.

Supplementary Figure 13

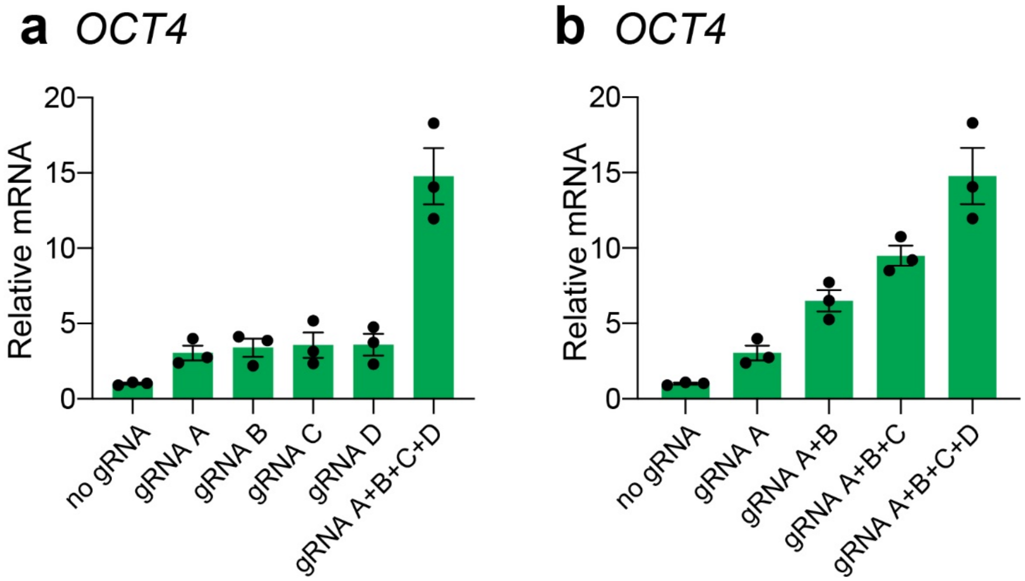

Supplementary Figure 13. dCas9-dMSK1 mediated gene activation from the *OCT4* promoter is dose-responsive. a, b. mRNA levels of *OCT4* were measured by RT-qPCR 72 hours after co-transfection of dCas9-dMSK1 and corresponding gRNAs or indicated gRNA combinations. n = 3 independent experiments; error bars, s.e.m.

Supplementary Figure 14

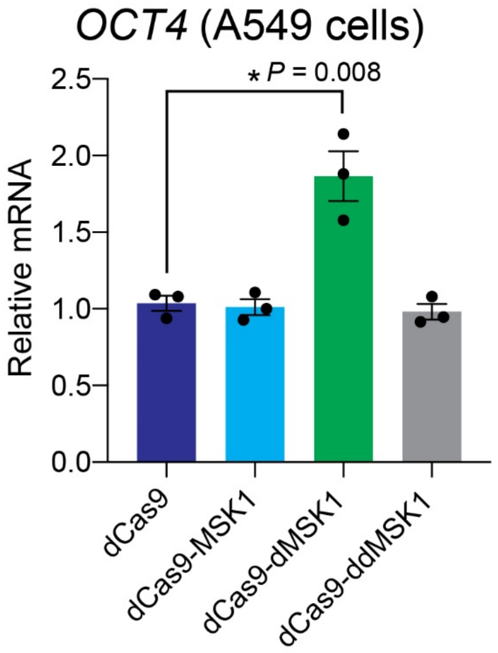

Supplementary Figure 14. dCas9-dMSK1 activates *OCT4* in A549 cells. *OCT4* mRNA was measured by RT-qPCR 72 hours after co-transfection of dCas9 or the indicated dCas9 fusions and gRNAs targeting the *OCT4* promoter in A549 cells. 2-sided *t*-test, $*P < 0.05$; n = 3 independent experiments; error bars, s.e.m.

Supplementary Figure 15

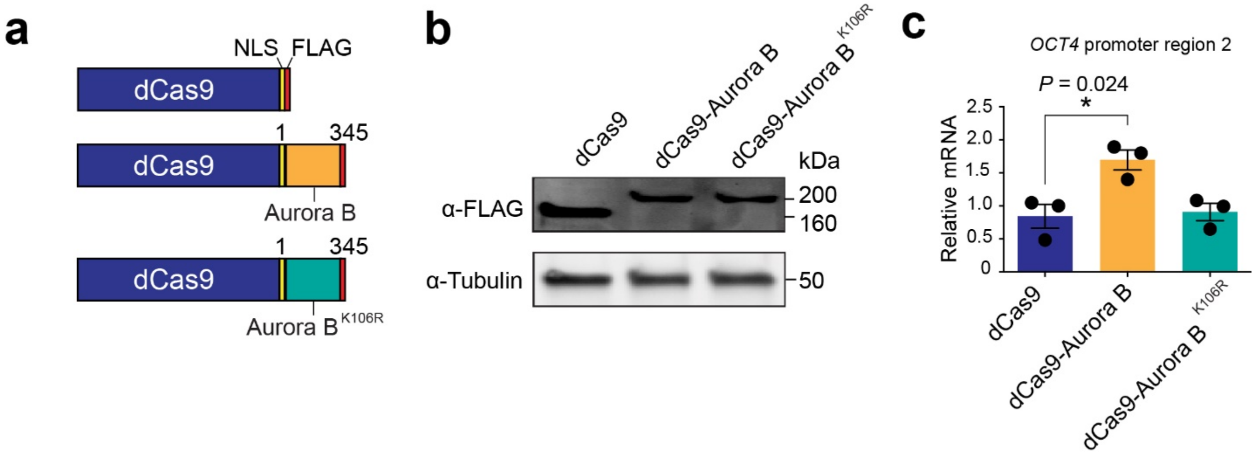

Supplementary Figure 15. dCas9-Aurora B activates gene expression from the *OCT4* promoter. a. Schematic of dCas9, dCas9-Aurora B, and a catalytically inactivated dCas9-Aurora B (harboring a K160R mutation). **b.** Plasmids encoding dCas9 or dCas9-Aurora B fusion proteins were transiently transfected into HEK293T cells and protein expression levels were detected 72 hours post-transfection by Western blotting with indicated antibodies. kDa, kilodaltons. Data are representative of 3 independent experiments. **c.** *OCT4* mRNA levels were measured using RT-qPCR 72 hours after co-transfection of dCas9 or indicated dCas9 fusion proteins and corresponding gRNAs. The pLV-dCas9-Aurora B-P2A-PuroR plasmid (dCas9-Aurora B) was generated by amplifying the Aurora B gene from cDNA sourced from HEK293T cells. The pLV-dCas9-Aurora B (K106R)-P2A-PuroR (dCas9- Aurora B^{K106R}) was created by amplification of the Aurora B gene from dCas9-Aurora B in overlapping fragments with primer sets designed to introduce the K106R mutation followed by cloning into the BamH1 digested pLV-dCas9-p300-P2A-Puro backbone (dCas9-P300, Addgene, #83889). 2-sided *t*-test, *$P < 0.05$; n = 3 independent experiments; error bars, s.e.m.; kDa, kilodaltons.

Supplementary Figure 16

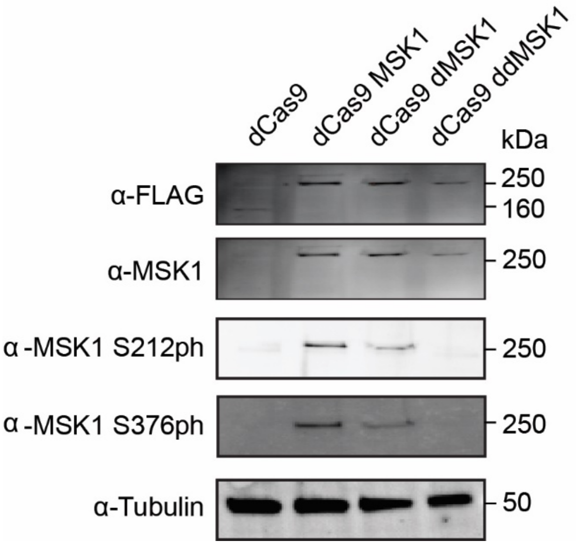

Supplementary Figure 16. dCas9-MSK1 fusions are expressed in A375 cells. Indicated dCas9-MSK1 fusion protein variants were delivered into A375 cells using lentiviral transduction. Protein expression and auto-phosphorylation levels were detected using Western blot. kDa, kilodaltons. Data are representative of 3 independent experiments.

Supplementary Figure 17

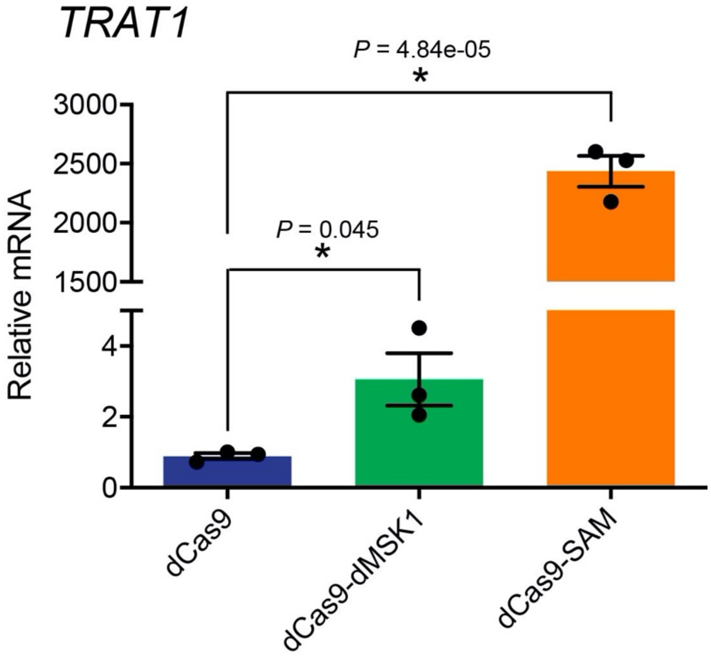

Supplementary Figure 17. *TRAT1* is activated by both dCas9-dMSK1 and dCas9-SAM. A375 cells were stably transduced with dCas9, dCas9-dMSK1, or dCas9-SAM and then transduced with a gRNA targeting the *TRAT1* promoter region. *TRAT1* mRNA was measured by RT-qPCR. 2-sided *t*-test, *$P < 0.05$; n = 3 independent experiments; error bars, s.e.m.

Supplementary Figure 18

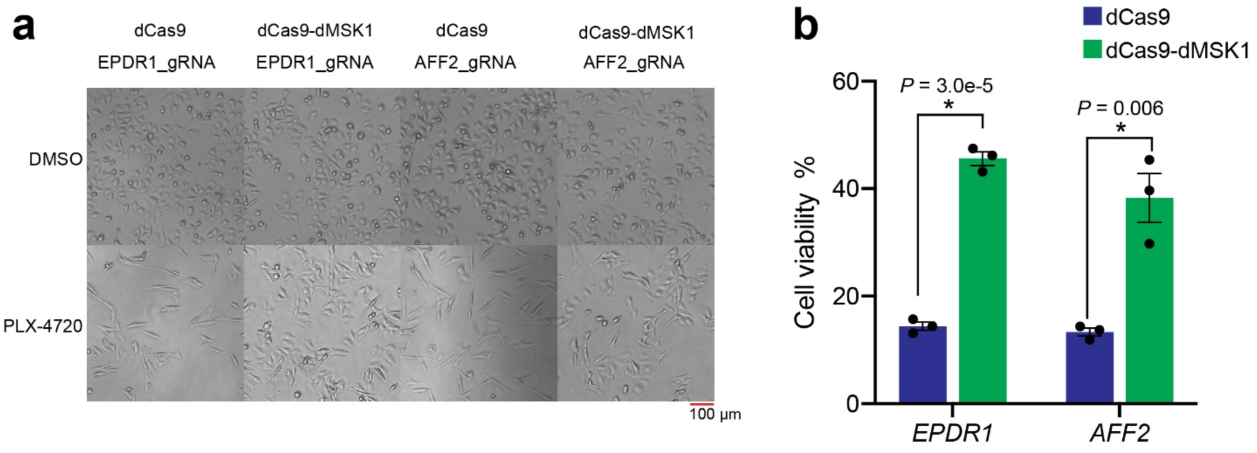

Supplementary Figure 18. dCas9-dMSK1-mediated upregulation of *EPDR1* or *AFF2* confers resistance to PLX-4720. a. A375 cells were stably transduced with dCas9 or dCas9-dMSK1 and indicated gRNAs, treated with DMSO (control) or PLX-4720, and then imaged to assess aberrant morphology. Data are representative of 3 independent experiments. **b.** A375 cells were stably transduced with dCas9 or dCas9-dMSK1 and indicated gRNAs, treated with DMSO (control) or PLX-4720 and then MTT assays were used to assess relative cell viability. 2-sided *t*-test, $^*P < 0.05$; n = 3 independent experiments; error bars, s.e.m.

Supplementary Table 1. Downregulated genes in MSK1 knockout HEK293T cell lines.*

WT vs KO			WT vs KO1			WT vs KO2		
Gene name	log2FC	*P*adj	Gene name	log2FC	*P*adj	Gene name	log2FC	*P*adj
CASC19	-10.57	2.65E-20	CASC19	-10.63	1.02E-10	*CASC19*	-10.49	3.01E-10
PRKCB	-3.74	7.53E-58	RNA5-8SN4	-7.98	1.37E-03	*TPTEP1*	-7.08	1.59E-08
BMP2	-3.23	2.98E-07	PRKCB	-3.51	2.47E-55	*SYNDIG1*	-6.08	3.58E-14
SHROOM2	-2.79	7.65E-13	RASL10B	-2.90	2.82E-56	*GSPT2*	-5.39	1.31E-21
AC004835.1	-2.30	2.81E-12	BMP2	-2.77	1.65E-63	*PRKCB*	-4.02	6.87E-52
ZNF462	-2.17	2.11E-12	GSPT2	-2.56	1.92E-14	*AC103702.2*	-4.00	8.95E-13
GDF6	-2.15	1.44E-12	SHROOM2	-2.44	6.68E-10	*BMP2*	-3.93	1.86E-69
HOXC12	-2.08	1.76E-07	IGFBPL1	-2.39	1.76E-14	*MID1*	-3.50	5.42E-12
XYLT1	-2.01	3.46E-16	FLT1	-2.21	8.04E-08	*SHROOM2*	-3.29	9.51E-13
C2CD4C	-2.00	2.47E-05	XYLT1	-2.19	2.24E-20	*OAS3*	-3.13	1.03E-09
UBE2QL1	-1.83	1.02E-04	ZNF462	-2.10	9.64E-09	*PXDN*	-2.71	1.78E-21
DUSP5	-1.80	2.45E-10	FOXF1	-2.08	1.07E-29	*AC004835.1*	-2.68	1.48E-10
FLT1	-1.69	1.18E-04	AC004835.1	-2.00	6.18E-08	*C2CD4C*	-2.62	3.45E-07
FOXF1	-1.65	4.54E-09	GDF6	-1.87	5.64E-09	*CDYL2*	-2.60	6.21E-03
ISM1	-1.53	1.85E-07	ZC3HAV1L	-1.78	3.09E-24	*UBE2QL1*	-2.57	8.58E-08
BARX2	-1.52	2.26E-08	DUSP5	-1.71	4.29E-08	*GDF6*	-2.52	3.31E-12
MIER2	-1.48	1.42E-06	C2CD4C	-1.57	1.29E-04	*ZNF462*	-2.23	8.49E-08
ZNF284	-1.45	1.35E-03	CDYL2	-1.49	5.90E-07	*FAM174B*	-1.95	2.30E-04
LXN	-1.44	1.02E-07	LRATD2	-1.42	9.93E-10	*DUSP5*	-1.90	3.27E-07
FOXO4	-1.39	6.99E-11	ZBED6CL	-1.40	2.04E-23	*HOXC12*	-1.89	5.70E-04
LRATD2	-1.37	6.86E-07	ISM1	-1.39	5.13E-05	*BARX2*	-1.89	2.58E-13
ZNF547	-1.35	1.81E-04	NDNF	-1.37	2.13E-07	*RPS6KA5*	-1.89	1.89E-05
B4GALNT4	-1.29	3.51E-04	HIST1H2BG	-1.36	1.39E-19	*XYLT1*	-1.84	4.02E-10
NDNF	-1.23	4.35E-06	MIER2	-1.36	3.64E-05	*ISM1*	-1.69	1.16E-05
HOTAIR	-1.22	4.68E-05	UBE2QL1	-1.35	1.03E-03	*LXN*	-1.67	2.67E-07
PIP4P2	-1.22	2.48E-04	AC011815.1	-1.35	6.02E-10	*MIER2*	-1.61	7.41E-05
AC004080.2	-1.18	9.52E-04	CGNL1	-1.33	3.80E-06	*HOTAIR*	-1.54	2.22E-06
FRRS1L	-1.14	3.01E-06	HIST1H2AE	-1.32	2.45E-17	*FOXO4*	-1.52	9.65E-11
ABCA1	-1.14	4.28E-05	MGAT3	-1.31	1.50E-08	*HSPA1B*	-1.47	8.53E-08
CLMN	-1.13	1.40E-04	NID2	-1.31	3.06E-11	*ZNF547*	-1.41	6.23E-03
FSTL1	-1.12	3.43E-09	FSTL1	-1.29	5.89E-26	*COL2A1*	-1.39	3.48E-03
EBF2	-1.11	4.08E-03	AC004080.2	-1.27	6.13E-04	*ZCCHC12*	-1.39	1.27E-17
SYT16	-1.09	2.21E-03	HAND1	-1.27	1.94E-12	*FOXF1*	-1.31	1.13E-08
WNT11	-1.07	8.67E-03	PFKFB4	-1.27	3.86E-06	*FLT1*	-1.29	8.00E-03
PMAIP1	-1.06	1.26E-06	CLMN	-1.26	2.43E-04	*SMAD6*	-1.28	1.06E-08
SLFN5	-1.06	7.27E-03	FOXO4	-1.26	1.25E-09	*NES*	-1.26	1.25E-04
ZBED6CL	-1.05	5.25E-05	LXN	-1.24	1.99E-05	*SLIT2*	-1.25	6.37E-14
CUL4B	-1.05	7.24E-06	FRRS1L	-1.23	3.15E-06	*IGFBPL1*	-1.23	3.32E-04
HOXA13	-1.03	2.21E-06	ABCA1	-1.23	2.51E-05	*EN1*	-1.17	7.40E-03
GPM6B	-1.02	8.43E-06	PIP4P2	-1.23	1.52E-03	*GPR176*	-1.17	3.67E-05
AMOT	-1.01	5.38E-05	BARX2	-1.22	5.13E-08	*SKIDA1*	-1.17	5.75E-03

Supplementary Table 1. Downregulated genes in MSK1 knockout HEK293T cell lines (continued).*

WT vs KO			WT vs KO1			WT vs KO2		
Gene name	log2FC	*P*adj	Gene name	log2FC	*P*adj	Gene name	log2FC	*P*adj
			HOXA13	-1.22	7.20E-16	*ACBD3*	-1.16	3.14E-12
			AMOT	-1.19	1.90E-13	*SAMD12*	-1.15	1.99E-04
			AL928654.4	-1.19	1.52E-03	*PDE4DIP*	-1.13	1.61E-06
			GDF7	-1.19	4.39E-10	*NDNF*	-1.09	1.44E-03
			GFPT2	-1.19	2.85E-06	*DTX3L*	-1.08	7.41E-05
			AMOT	-1.17	6.53E-10	*EMILIN2*	-1.08	1.11E-03
			ZNF888	-1.17	1.47E-09	*SMAD6*	-1.06	1.26E-03
			PMAIP1	-1.16	1.23E-10	*TSPAN3*	-1.05	3.82E-05
			GPM6B	-1.14	2.45E-07	*RHOU*	-1.05	1.49E-07
			PPP1R3G	-1.13	9.44E-03	*PLXNA2*	-1.05	1.37E-03
			CUL4B	-1.13	1.02E-09	*FRRS1L*	-1.04	1.23E-03
			B4GALNT4	-1.13	1.29E-04	*ABCA1*	-1.03	5.70E-03
			AMOT	-1.10	7.68E-05	*MSX2*	-1.03	8.21E-05
			LOXL2	-1.10	4.52E-04	*RAB31*	-1.01	2.46E-08
			FZD4	-1.08	1.20E-10	*ILDR2*	-1.00	4.82E-05
			SPRY2	-1.06	7.56E-04			
			PREX2	-1.05	2.15E-03			
			FUT11	-1.05	8.62E-03			
			ZNF536	-1.04	3.71E-03			
			PXDN	-1.04	3.58E-08			
			SCD5	-1.03	3.09E-07			
			KREMEN1	-1.01	6.58E-12			
			EFNA2	-1.01	2.39E-03			
			TRIM56	-1.00	5.97E-04			

*Data was analyzed using the Wald test and the adjusted *P* value (P_{adj}) was calculated using the Benjamini and Hochberg method.

Supplementary Table 2. **Upregulated genes in MSK1 knockout HEK293T cell lines.***

WT vs KO			WT vs KO1			WT vs KO2		
Gene name	log2FC	*P*adj	Gene name	log2FC	*P*adj	Gene name	log2FC	*P*adj
NWD2	8.95	4.74E-07	*KRTAP19-1*	3.96	3.59E-52	*BEX1*	6.24	3.35E-131
BEX1	5.50	9.02E-07	*BEX1*	3.91	8.95E-39	*MAGEB2*	5.03	6.73E-14
KRTAP19-1	3.28	8.67E-03	*RPRM*	2.76	7.09E-19	*RPRM*	3.18	9.55E-21
RPRM	2.98	1.43E-23	*TCF24*	2.26	5.01E-15	*GPR50*	2.89	2.59E-23
NPY1R	2.68	5.24E-13	*GPR50*	2.01	7.37E-12	*AL035446.1*	2.82	1.98E-20
LRFN5	2.53	5.66E-07	*RAB9B*	1.44	2.39E-10	*LINC01694*	2.67	6.73E-14
GPR50	2.51	2.82E-14	*TMSB15A*	1.34	3.02E-08	*CELSR3*	2.59	9.37E-05
TCF24	2.42	1.95E-20	*PITHD1*	1.32	2.84E-03	*TCF24*	2.57	1.04E-16
CCND1	1.74	4.35E-06	*LINC00630*	1.31	4.76E-04	*AL035446.2*	2.56	2.15E-12
SLC25A53	1.72	2.92E-07	*BCAP31*	1.27	1.06E-03	*KRTAP19-1*	2.05	2.28E-04
RAB3C	1.67	2.92E-07	*HIST1H4I*	1.24	1.76E-11	*MDGA2*	1.85	3.96E-04
MDGA2	1.64	1.04E-04	*HIST2H2BC*	1.20	1.38E-03	*SLC25A53*	1.82	1.12E-04
AHCTF1	1.52	3.94E-03	*FAM120C*	1.18	2.46E-05	*CCND1*	1.81	6.54E-04
ARMCX5-GPRASP2	1.40	1.91E-04	*CELSR3*	1.18	1.00E-05	*BEX4*	1.63	4.56E-06
RAB9B	1.34	4.41E-10	*GPR37*	1.16	2.39E-03	*RAB3C*	1.59	1.28E-03
BEX4	1.33	4.43E-04	*ZNF672*	1.08	1.13E-08	*ARMCX5-GPRASP2*	1.58	1.72E-03
GALNT13	1.32	3.53E-06	*LARP1*	1.07	4.51E-03	*GALNT13*	1.56	8.58E-08
LINC00630	1.24	1.47E-04	*ZNF695*	1.06	4.90E-03	*RNF128*	1.46	2.27E-05
ZNF672	1.23	5.82E-09	*LDOC1*	1.05	3.50E-03	*GUCY1A2*	1.38	2.67E-03
TMSB15A	1.20	8.04E-07	*GALNT13*	1.05	8.57E-04	*ZNF672*	1.37	5.43E-10
TAS1R3	1.20	8.67E-03				*BCRP2*	1.28	5.03E-03
RNF128	1.19	4.89E-04				*BSN*	1.28	3.85E-05
LDOC1	1.14	5.88E-05				*LDOC1*	1.24	7.20E-04
FGF9	1.13	9.17E-04				*RAB9B*	1.24	1.16E-05
						AC097639.1	1.24	5.28E-03
						FH	1.19	9.88E-13
						NPTX1	1.17	1.67E-06
						RTL8A	1.17	1.68E-03
						SCCPDH	1.15	8.99E-10
						EPHA3	1.11	8.04E-05
						RPL29	1.08	2.23E-07
						CCDC85B	1.07	1.54E-04
						ZNF670	1.05	4.99E-04
						TMSB15A	1.05	6.75E-04
						RPL10P9	1.05	5.02E-04
						STT3B	1.01	5.03E-03

*Data was analyzed using the Wald test and the adjusted P value (P_{adj}) was calculated using the Benjamini and Hochberg method.

Supplementary Table 3. RT-qPCR Ct values for genes tested in HEK293T cells.

Gene Name	Basal Ct	Activated Ct
PRKCB	26 ± 1	26 ± 1
BMP2	27 ± 1	25 ± 1
SHOOM2	29 ± 1	29 ± 1
GDF6	30 ± 1	28 ± 1
ZNF462	27 ± 1	27 ± 1
OCT4	30 ± 1	26 ± 1
MYOD	31 ± 1	29 ± 1
HBA1	25 ± 1	24 ± 1
SOX2	24 ± 1	23 ± 1
KLF4	25 ± 1	24 ± 1

Supplementary Table 4. Top 100 enriched gRNAs from genome-scale dCas9-dMSK1 screen.*

Gene name	log2 Fold Change	P value
EPDR1	9.07	4.39E-09
AFF2	8.91	8.17E-10
TRAT1	8.84	4.51E-11
MRPS15	8.74	1.18E-07
ERC2	8.51	6.86E-05
LACC1	8.47	7.09E-07
AGL	8.39	1.42E-08
TDRP	8.26	5.96E-07
MIPOL1	8.19	2.49E-06
LELP1	8.16	1.00E-05
KAZN	8.15	1.05E-04
PROKR2	8.10	2.25E-07
C9orf89	8.08	3.74E-08
WNT7A	7.96	1.23E-04
PRICKLE2	7.55	1.67E-06
ARMCX5-GPRASP2	7.50	4.37E-08
WDR4	7.45	1.44E-05
LCE1E	7.40	4.62E-06
SKIV2L	7.32	1.92E-06
GGT1	7.13	1.87E-06
PRKAA1	7.11	5.61E-04
FGFR2	7.05	1.69E-06
SERPINF1	6.66	4.00E-06
CHST8	6.30	1.17E-06
LMNA	6.03	1.18E-06
MARK4	5.44	8.80E-04
RBM45	5.42	2.93E-04
LOC154872	5.29	5.29E-05
EXOC7	5.08	2.43E-04
KRT10	4.96	9.64E-03
MCF2L	4.95	1.70E-03
RAD18	4.84	9.93E-05
HCAR3	4.77	5.59E-04
PLCL2	4.72	5.89E-03
PSG5	4.71	3.30E-03
ICAM1	4.60	2.42E-04
ARHGDIG	4.54	3.49E-04
ZFAND6	4.50	4.97E-05
SLC6A18	4.48	1.58E-05
GAGE1	4.43	1.09E-06

SHISA5	4.31	4.57E-05
NDUFAF3	4.30	4.19E-06
AGO2	4.22	4.57E-04
RNF19A	4.22	2.91E-05
PIH1D1	4.21	3.70E-04
AK8	4.03	2.08E-03
SPIN2A	4.01	1.31E-04
TRIM59	4.01	1.47E-04
DUX4L2	4.01	9.56E-06
AK4	3.94	6.16E-05
TCF7	3.84	1.82E-04
PTGES3	3.84	9.74E-03
LAMP2	3.80	8.77E-04
CREBBP	3.77	1.86E-03
OR2F1	3.70	1.43E-04
CYP2C19	3.67	1.03E-04
SCN5A	3.62	2.74E-03
MPV17L	3.58	6.32E-03
LYPD1	3.56	3.10E-05
ETS1	3.51	4.49E-05
AP5S1	3.49	4.13E-05
SOX21	3.49	3.92E-03
RAI2	3.48	2.43E-03
YIPF7	3.46	1.07E-03
KRTAP4-3	3.46	7.39E-03
ALG13	3.43	1.30E-03
FAM71D	3.38	2.89E-05
GPR33	3.36	5.12E-06
FBXO39	3.30	4.79E-04
ICAM2	3.30	1.15E-03
MAPKAP1	3.28	2.41E-03
CD63	3.27	3.48E-03
EFNA1	3.26	4.59E-05
KCNT1	3.26	2.79E-03
CDK11B	3.26	2.63E-03
LMOD1	3.26	1.10E-03
RGS12	3.25	5.88E-03
ATP6V1B2	3.24	2.16E-03
CSMD2	3.19	9.98E-07
PSIP1	3.18	1.08E-04
RSPRY1	3.14	2.79E-05
PALB2	3.14	4.70E-04
RAET1L	3.13	1.49E-03

SETD4	3.12	3.01E-05
DDX56	3.11	3.73E-06
USP9Y	3.08	6.28E-05
CYP51A1	3.03	6.09E-04
CLDN7	3.02	3.38E-04
ARHGEF19	3.00	4.87E-04
SLC7A7	3.00	3.45E-03
ZC3HC1	2.99	1.85E-03
KLHL13	2.93	2.88E-05
GATM	2.93	6.74E-04
NRG4	2.93	8.42E-03
VEPH1	2.90	1.89E-04
TBRG4	2.90	6.69E-03
HSFY2	2.89	3.72E-04
NTRK3	2.89	1.12E-04
TCTEX1D1	2.87	7.18E-04
ANKMY1	2.86	9.99E-04

*Data was analyzed using robust rank aggregation (RRA).

646 **Supplementary Table 5. gRNAs used in this study.**

Target Location	Protospacer Sequence (5'- 3')	Genomic Location (GRCh38/hg38 Assembly)	Reference
OCT4_PE_A	ACTTCAGGTTCAAAGAAGCC	chr6: 31171948-31171967	Hilton et al, Nat. Biotech. 2015
OCT4_PE_B	CCCTGGGTGGGGAAAACCAG	chr6: 31171898 31171917	Hilton et al, Nat. Biotech. 2015
OCT4_PE_C	TTTTCCCCACCCAGGGCCTA	chr6: 31171894 31171913	Hilton et al, Nat. Biotech. 2015
OCT4_PE_D	AGGGAGAACGGGGCCTACCG	chr6: 31171843 31171862	Hilton et al, Nat. Biotech. 2015
OCT4_PE_E	CAGACATCTAATACCACGGT	chr6: 31171827 31171846	Hilton et al, Nat. Biotech. 2015
OCT4_PE_F	AGTGATAAGACACCCGCTTT	chr6: 31171747 31171766	Hilton et al, Nat. Biotech. 2015
OCT4_DE_A	GCATGACAAAGGTGCCGTGA	chr6: 31173098 31173117	Hilton et al, Nat. Biotech. 2015
OCT4_DE_B	GTGCCGTGATGGTTCTGTCC	chr6: 31173087 31173106	Hilton et al, Nat. Biotech. 2015
OCT4_DE_C	GGAGGAACATGCTTCGGAAC	chr6: 31173032 31173051	Hilton et al, Nat. Biotech. 2015
OCT4_DE_D	CCTGCCTTTTGGGCAGTTAA	chr6: 31172987 31173006	Hilton et al, Nat. Biotech. 2015
OCT4_DE_E	TCGGCCTTTAACTGCCCAAA	chr6: 31172980 31172999	Hilton et al, Nat. Biotech. 2015
OCT4_DE_F	GGTCTGCCGGAAGGTCTACA	chr6: 31172930 31172949	Hilton et al, Nat. Biotech. 2015
OCT4_PP_Region1_A	CAGAGCCTGAGAGAGTGCCA	chr6: 31171212 31171231	This study
OCT4_PP_Region1_B	CACGCCTTTAATCATGACAC	chr6: 31171154 31171173	This study
OCT4_PP_Region1_C	AAAAAACTAACAGGGCACAG	chr6: 31171046 31171065	This study
OCT4_PP_Region1_D	TGCTTTGGCCCAGTAGATCG	chr6: 31170977 31170996	This study
OCT4_PP_Region1_E	TCAATGTAGCCTCGATCTAC	chr6: 31170965 31170984	This study
OCT4_PP_Region2_A	ACTCCACTGCACTCCAGTCT	chr6: 31138711-31138730	Hu et al., Nucleic Acids Res., 2014
OCT4_PP_Region2_B	TCTGTGGGGGACCTGCACTG	chr6: 31138643-31138662	Hu et al., Nucleic Acids Res., 2014
OCT4_PP_Region2_C	GGGGCGCCAGTTGTGTCTCC	chr6: 31138613-31138632	Hu et al., Nucleic Acids Res., 2014
OCT4_PP_Region2_D	ACACCATTGCCACCACCATT	chr6: 31138574-31138593	Hu et al., Nucleic Acids Res., 2014
OCT4_PP_Region3_A	CTCTCCTCCACCCATCCAGG	chr6: 31170738 31170757	This study
OCT4_PP_Region3_B	CACCCACTAGCCTTGACCTC	chr6: 31170707-31170726	This study
OCT4_PP_Region3_C	TCGCAAGCCCTCATTTCACC	chr6: 31170652 31170671	This study
OCT4_PP_Region3_D	GAAGCCAGGTGTCCCGCCAT	chr6: 31170601 31170620	This study
OCT4_PP_Region3_E	ATCACCTCCACCACCTGGAG	chr6: 31170561 31170580	This study
MYOD_DRR_A	GGCTGGATTGGGTTTCCAG	chr11:17736065-17736083	Hilton et al, Nat. Biotech. 2015
MYOD_DRR_B	GGCCCACATTCCTTTCCAG	chr11:17736158-17736176	Hilton et al, Nat. Biotech. 2015
MYOD_DRR_C	CATGAAGACAGCAGAAGCC	chr11:17736311-17736329	Hilton et al, Nat. Biotech. 2015
MYOD_DRR_D	TGTTTTCAGCTTCCAAACT	chr11:17736528-17736546	Hilton et al, Nat. Biotech. 2015
MYOD_CE_A	CTTCCTATAAACTTCTGAG	chr11:17721139-17721157	Hilton et al, Nat. Biotech. 2015
MYOD_CE_B	CAGCAGCTGGTCACAAAGC	chr11:17721200-17721218	Hilton et al, Nat. Biotech. 2015
MYOD_CE_C	CTCACAGCACAGCCAGTGT	chr11:17721257-17721275	Hilton et al, Nat. Biotech. 2015
MYOD_CE_D	CAACTGAGTCCTGAGGTTT	chr11:17721347-17721365	Hilton et al, Nat. Biotech. 2015
MYOD_promoter_Region1_A	GCAGAAAGCTTAGAGCGTCA	chr11:17719043 17719062	This study
MYOD_promoter_Region1_B	CGTGAAAGAGGGACCGAAAG	chr11:17719084 17719103	This study
MYOD_promoter_Region1_C	TTGGAGAGAGGCAAGGCATT	chr11:17719130 17719149	This study
MYOD_promoter_Region1_D	TCTACAGCCGCTCTACCCAT	chr11:17719168 17719187	This study
MYOD_promoter_Region1_E	AGTATTGAAAGTCAGCTCAG	chr11:17719216 17719235	This study

MYOD_promoter_Region2_A	CCTGGGCTCCGGGGCGTTTAGG	chr11:17719509 17719530	Perez-Pinera et al., Nat. Methods, 2013
MYOD_promoter_Region2_B	GGCCCCTGCGGCCACCCCGGG	chr11:17719422 17719442	Perez-Pinera et al., Nat. Methods, 2013
MYOD_promoter_Region2_C	CTCCCTCCCTGCCCGGTAGGG	chr11:17719350 17719370	Perez-Pinera et al., Nat. Methods, 2013
MYOD_promoter_Region2_D	AGGTTTGGAAAGGGCGTGCC	chr11:17719290 17719309	Perez-Pinera et al., Nat. Methods, 2013
MYOD_promoter_Region3_A	AACCACAAATCAGGCCGGAC	chr11:17719611 17719630	This study
MYOD_promoter_Region3_B	GGCTGTAGATAGCAAAGTGC	chr11:17719677 17719696	This study
MYOD_promoter_Region3_C	CTGGCTTCGCCCAACCCAAG	chr11:17719738 17719757	This study
MYOD_promoter_Region3_D	GCGACAGTAGCTCCATATCC	chr11:17719779 17719798	This study
PRKCB_promoter_A	AGCGAGAGTGGAAAGACCTA	chr16: 23835682 23835701	This study
PRKCB_promoter_B	AGGCTCGGGTCCGACGACCC	chr16:23835766 23835785	This study
PRKCB_promoter_C	TGCTTTACATATCGGCGCCC	chr16:23835853 23835872	This study
PRKCB_promoter_D	GGCACCGCTGGCCCCAGCTG	chr16:23835953 23835972	This study
BMP2_promoter_A	CTCTCAAAGGAGAGATCAGC	chr20: 6767441 6767460	This study
BMP2_promoter_B	CCCTCCGCCCCACCCCGCCG	chr20:6767508 6767527	This study
BMP2_promoter_C	CCGGCGAGCCGCGCCGCGAA	chr20:6767587 6767606	This study
BMP2_promoter_D	AAGCCGCGAGCGCCGCGCCA	chr20:6767662 6767681	This study
SHROOM2_promoter_A	GCGTCCAGAGGTCCAGCCCG	chrX:9786253 9786272	This study
SHROOM2_promoter_B	GCGCCCGCCCCGCCCCCCGT	chrX:9786311 9786330	This study
SHROOM2_promoter_C	CCCCCTCCCCGCCAGCCCTC	chrX:9786383 9786402	This study
SHROOM2_promoter_D	TTGGAAAGAAAGTGCCGGCC	chrX:9786422 9786441	This study
GDF6_promoter_A	GGCACGGAGCGGCTGGACAG	chr8: 96160750 96160769	This study
GDF6_promoter_B	GGTCCCAGCCACACAAACCC	chr8:96160816 96160835	This study
GDF6_promoter_C	CCTCCCCATTCCTCCCCTCC	chr8:96160869 96160888	This study
GDF6_promoter_D	CCTGCCACACCGGCGCACCG	chr8:96160964 96160983	This study
ZNF462_promoter_A	TCTCCTGCCACACAAAACGC	chr9: 106862919 106862938	This study
ZNF462_promoter_B	TCTCTGAATAATGAGCAACC	chr9:106862985 106863004	This study
ZNF462_promoter_C	GGGAGGAGAGGAGAGAGAAG	chr9:106863047 106863066	This study
ZNF462_promoter_D	GGGAGAGAGACGGATATCTC	chr9:106863126 106863145	This study
EPDR1_promoter	GTCGAGGGGCAATGTAGGCG	chr7: 37920406 37920425	This study
MRPS15_promoter	TCCGTCCTTAATCTCATTCC	chr1: 36464582 36464601	This study
TRAT1_promoter	ATAAAGGTAGGAATTAAACC	chr3: 108822557 108822576	This study
AFF2_promoter	GTTTGATAGTTTGAGTATTC	chrX: 148718929 148718948	This study
ERC2_promoter	GCGCGTCTCGGGACTGCGGC	chr3: 56468506 56468525	This study
TDRP_promoter	GAGGCGCCCACAGATTCCTG	chr8: 545923 545942	This study
LACC1_promoter	ACTTCAGCTCGCTTGGGAAA	chr13: 43879102 43879121	This study
MIPOL1_promoter	GCGTTCTGCGCTCGTCGTCT	chr14: 37197714 37197733	This study
AGL_promoter	TGGGGAAGGTGCACCGGCCA	chr1: 99849934 99849953	This study
LELP1_promoter	TGGCTCTATTCTGGTGTTCA	chr1: 153203300 153203319	This study
MSK1_ko1	TCACTGTCAAGCACGAGCTG	chr14: 91060339 91060358	This study
MSK1_ko2	CTCCTGAAGGTCCTAGGAAC	chr14: 91001093 91001112	This study
Non-targeting gRNA	GTATTACTGATATTGGTGGG	none	Doench et al, Nat. Biotech. 2016

Supplementary Table 6. Quantitative reverse transcription PCR and ChIP-qPCR primers and conditions.

Target	Forward Primer (5’-3’)	Reverse Primer (5’-3’)	Cycling Parameters	
GAPDH	CAATGACCCCTTCATTGACC	TTGATTTTGGAGGGATCTCG	95˚C 30 sec<br>95˚C 10 sec<br>60˚C 30 sec	40 x
OCT4	CGAAAGAGAAAGCGAACCAGTATCGAGAAC	CGTTGTGCATAGTCGCTGCTTGATCGC	95˚C 30 sec<br>95˚C 10 sec<br>60˚C 30 sec	40 x
MYOD	CTCTCTGCTCCTTTGCCACA	GTGCTCTTCGGGTTTCAGGA	95˚C 30 sec<br>95˚C 10 sec<br>60˚C 30 sec	40 x
PRKCB	GTTGTGATCCAAGATGATGACG	CTGTAAGAAGAACAGACCGATGG	95˚C 30 sec<br>95˚C 10 sec<br>60˚C 30 sec	40 x
BMP2	TGTATCGCAGGCACTCAGGTCA	CCACTCGTTTCTGGTAGTTCTTC	95˚C 30 sec<br>95˚C 10 sec<br>60˚C 30 sec	40 x
SHROOM2	CCTGAAGCTGGTCGTCAAAAG	CGCTGTAGGTTCGTCTGCTCCC	95˚C 30 sec<br>95˚C 10 sec<br>60˚C 30 sec	40 x
GDF6	GGACTTACTCCATCGCTGAGAAG	GGAGAGGAGTGTGCGAGAGATC	95˚C 30 sec<br>95˚C 10 sec<br>60˚C 30 sec	40 x
ZNF462	GCAATCACCTCCGAAAGCACGT	AACAAACGAGCAATACTGGCAGC	95˚C 30 sec<br>95˚C 10 sec<br>60˚C 30 sec	40 x
EPDR1	CGGACAGAAAGTCAGCTAGATCC	CGCGTAGACAATATCACACTGTAG	95˚C 30 sec<br>95˚C 10 sec<br>60˚C 30 sec	40 x
MRPS15	CCGATTCGTGACCAAGAAGGCT	TCTGGGTTCCTCCGCTTTGCTT	95˚C 30 sec<br>95˚C 10 sec<br>60˚C 30 sec	40 x
TRAT1	ACAGCTACTCCAGTGACCACAC	CAGATTTCTCTGGTCGGGCTTTC	95˚C 30 sec<br>95˚C 10 sec<br>60˚C 30 sec	40 x
AFF2	CTTCCCAGTGATCCAAGCTGTG	GCTCAGAGTGTCCAGCAGTATG	95˚C 30 sec<br>95˚C 10 sec<br>60˚C 30 sec	40 x
ERC2	CAAGACGAAGGCTCTCCAGACT	CAGTGTTCAGCACACCATTGGC	95˚C 30 sec<br>95˚C 10 sec<br>60˚C 30 sec	40 x
TDRP	CGAGGTTGGAAAGAAGTGACTTC	CCAGATTTCTTCTCTGCCTTCAAC	95˚C 30 sec<br>95˚C 10 sec<br>60˚C 30 sec	40 x
LACC1	ATCTGCGTAGGTTGGCGAATGC	AGAGTCAGGCTCCTTTCTTCCC	95˚C 30 sec<br>95˚C 10 sec<br>60˚C 30 sec	40 x
MIPOL1	ACTGCAACAAGCTCTGACAGAGC	CTCAGTACATCCACCAGCCTTTC	95˚C 30 sec<br>95˚C 10 sec<br>60˚C 30 sec	40 x
LELP1	AGTGTGAGTCCAAATGCCAGCC	CAGGATGAAGGAGACTGCGAGG	95˚C 30 sec<br>95˚C 10 sec<br>60˚C 30 sec	40 x

AGL	CAAGCTGGAGTTGCCACAAAAGG	CAACAGCGACTTGTGCATGTGG	95˚C 30 sec<br>95˚C 10 sec<br>60˚C 30 sec	40 x
HBA1	GTATGGTGCGGAGGCCCTGGAG	ACAGCGCGTTGGGCATGTCGTC	95˚C 30 sec<br>95˚C 10 sec<br>60˚C 30 sec	40 x
SOX2	GCTACAGCATGATGCAGGACCA	TCTGCGAGCTGGTCATGGAGTT	95˚C 30 sec<br>95˚C 10 sec<br>60˚C 30 sec	40 x
KLF4	CCGCTCCATTACCAAG	CACGATCGTCTTCCCCTCTT	95˚C 30 sec<br>95˚C 10 sec<br>60˚C 30 sec	40 x
OCT4 ChIP-qPCR	GTCTGGGCAACAAAGTGAGA	CAATGGTGTCTGTGGAAGGG	95˚C 30 sec<br>95˚C 10 sec<br>60˚C 30 sec	40 x
MYOD ChIP-qPCR	GCAGCCGCCTAGGGCTGCCGGTCG	CCGAGGCCAATAGGAACACTGCGG	95˚C 30 sec<br>95˚C 10 sec<br>60˚C 30 sec	40 x
PRKCB ChIP-qPCR	CCGTAGGTCTTTCCACTCTC	GCTGCCAGCTGCTTTACATA	95˚C 30 sec<br>95˚C 10 sec<br>60˚C 30 sec	40 x
BMP2 ChIP-qPCR	CCTGCATTTGTCCTGGATTT	CCGAACACCTCCCCCTTC	95˚C 30 sec<br>95˚C 10 sec<br>60˚C 30 sec	40 x
SHROOM2 ChIP-qPCR	GTACGAGCTCCCGAACG	CGCCGTAACTTGGAAAGAAAG	95˚C 30 sec<br>95˚C 10 sec<br>60˚C 30 sec	40 x
GDF6 ChIP-qPCR	GGTCCCAGCCACACAAA	CGTCTTCCTGCCACACC	95˚C 30 sec<br>95˚C 10 sec<br>60˚C 30 sec	40 x
ZNF462 ChIP-qPCR	CTCTCTTTCTGTCTTGCTTTCT	TCTCCTCTTCTCTCTCCTCT	95˚C 30 sec<br>95˚C 10 sec<br>60˚C 30 sec	40 x

Supplementary Note 1. Amino acid sequences of dCas9 constructs.

dCas9: amino acid sequence; 1 X "Flag" Epitope, Nuclear Localization Sequence, Streptococcus pyogenes Cas9 (D10A, H840A).

MDKKYSIGLAIGTNSVGWAVITDEYKVPSKKFKVLGNTDRHSIKKNLIGALLFDSGETA
EATRLKRTARRRYTRRKNRICYLQEIFSNEMAKVDDSFFHRLEESFLVEEDKKHERHPIF
GNIVDEVAYHEKYPTIYHLRKKLVDSTDKADLRLIYLALAHMIKFRGHFLIEGDLNPDNS
DVDKLFIQLVQTYNQLFEENPINASGVDAKAILSARLSKSRRLENLIAQLPGEKKNGLFG
NLIALSLGLTPNFKSNFDLAEDAKLQLSKDTYDDDLDNLLAQIGDQYADLFLAAKNLSD
AILLSDILRVNTEITKAPLSASMIKRYDEHHQDLTLLKALVRQQLPEKYKEIFFDQSKNGY
AGYIDGGASQEEFYKFIKPILEKMDGTEELLVKLNREDLLRKQRTFDNGSIPHQIHLGEL
HAILRRQEDFYPFLKDNREKIEKILTFRIPYYVGPLARGNSRFAWMTRKSEETITPWNFEE
VVDKGASAQSFIERMTNFDKNLPNEKVLPKHSLLYEYFTVYNELTKVKYVTEGMRKPA
FLSGEQKKAIVDLLFKTNRKVTVKQLKEDYFKKIECFDSVEISGVEDRFNASLGTYHDLL
KIIKDKDFLDNEENEDILEDIVLTLTLFEDREMIEERLKTYAHLFDDKVMKQLKRRRYTG
WGRLSRKLINGIRDKQSGKTILDFLKSDGFANRNFMQLIHDDSLTFKEDIQKAQVSGQG
DSLHEHIANLAGSPAIKKGILQTVKVVDELVKVMGRHKPENIVIEMARENQTTQKGQKN
SRERMKRIEEGIKELGSQILKEHPVENTQLQNEKLYLYYLQNGRDMYVDQELDINRLSD
YDVDAIVPQSFLKDDSIDNKVLTRSDKNRGKSDNVPSEEVVKKMKNYWRQLLNAKLIT
QRKFDNLTKAERGGLSELDKAGFIKRQLVETRQITKHVAQILDSRMNTKYDENDKLIRE
VKVITLKSKLVSDFRKDFQFYKVREINNYHHAHDAYLNAVVGTALIKKYPKLESEFVYG
DYKVYDVRKMIAKSEQEIGKATAKYFFYSNIMNFFKTEITLANGEIRKRPLIETNGETGEI
VWDKGRDFATVRKVLSMPQVNIVKKTEVQTGGFSKESILPKRNSDKLIARKKDWDPKK
YGGFDSPTVAYSVLVVAKVEKGKSKKLKSVKELLGITIMERSSFEKNPIDFLEAKGYKE
VKKDLIIKLPKYSLFELENGRKRMLASAGELQKGNELALPSKYVNFLYLASHYEKLKGS
PEDNEQKQLFVEQHKHYLDEIIEQISEFSKRVILADANLDKVLSAYNKHRDKPIREQAENI
IHLFTLTNLGAPAAFKYFDTTIDRKRYTSTKEVLDATLIHQSITGLYETRIDLSQLGGDKR
PAATKKAGQAKKKKDYKDDDDK

dCas9-MSK1, amino acid sequence; 1 X "Flag" Epitope, Nuclear Localization Sequence, Streptococcus pyogenes Cas9 (D10A, H840A), human MSK1 amino acid sequence

MDKKYSIGLAIGTNSVGWAVITDEYKVPSKKFKVLGNTDRHSIKKNLIGALLFDSGETA
EATRLKRTARRRYTRRKNRICYLQEIFSNEMAKVDDSFFHRLEESFLVEEDKKHERHPIF
GNIVDEVAYHEKYPTIYHLRKKLVDSTDKADLRLIYLALAHMIKFRGHFLIEGDLNPDNS
DVDKLFIQLVQTYNQLFEENPINASGVDAKAILSARLSKSRRLENLIAQLPGEKKNGLFG
NLIALSLGLTPNFKSNFDLAEDAKLQLSKDTYDDDLDNLLAQIGDQYADLFLAAKNLSD
AILLSDILRVNTEITKAPLSASMIKRYDEHHQDLTLLKALVRQQLPEKYKEIFFDQSKNGY
AGYIDGGASQEEFYKFIKPILEKMDGTEELLVKLNREDLLRKQRTFDNGSIPHQIHLGEL
HAILRRQEDFYPFLKDNREKIEKILTFRIPYYVGPLARGNSRFAWMTRKSEETITPWNFEE
VVDKGASAQSFIERMTNFDKNLPNEKVLPKHSLLYEYFTVYNELTKVKYVTEGMRKPA
FLSGEQKKAIVDLLFKTNRKVTVKQLKEDYFKKIECFDSVEISGVEDRFNASLGTYHDLL
KIIKDKDFLDNEENEDILEDIVLTLTLFEDREMIEERLKTYAHLFDDKVMKQLKRRRYTG
WGRLSRKLINGIRDKQSGKTILDFLKSDGFANRNFMQLIHDDSLTFKEDIQKAQVSGQG
DSLHEHIANLAGSPAIKKGILQTVKVVDELVKVMGRHKPENIVIEMARENQTTQKGQKN
SRERMKRIEEGIKELGSQILKEHPVENTQLQNEKLYLYYLQNGRDMYVDQELDINRLSD
YDVDAIVPQSFLKDDSIDNKVLTRSDKNRGKSDNVPSEEVVKKMKNYWRQLLNAKLIT

QRKFDNLTKAERGGLSELDKAGFIKRQLVETRQITKHVAQILDSRMNTKYDENDKLIRE
VKVITLKSKLVSDFRKDFQFYKVREINNYHHAHDAYLNAVVGTALIKKYPKLESEFVYG
DYKVYDVRKMIAKSEQEIGKATAKYFFYSNIMNFFKTEITLANGEIRKRPLIETNGETGEI
VWDKGRDFATVRKVLSMPQVNIVKKTEVQTGGFSKESILPKRNSDKLIARKKDWDPKK
YGGFDSPTVAYSVLVVAKVEKGKSKKLKSVKELLGITIMERSSFEKNPIDFLEAKGYKE
VKKDLIIKLPKYSLFELENGRKRMLASAGELQKGNELALPSKYVNFLYLASHYEKLKGS
PEDNEQKQLFVEQHKHYLDEIIEQISEFSKRVILADANLDKVLSAYNKHRDKPIREQAENI
IHLFTLTNLGAPAAFKYFDTTIDRKRYTSTKEVLDATLIHQSITGLYETRIDLSQLGGDKR
PAATKKAGQAKKKKGSMEEEGGSSGGAAGTSADGGDGGEQLLTVKHELRTANLTGHA
EKVGIENFELLKVLGTGAYGKVFLVRKISGHDTGKLYAMKVLKKATIVQKAKTTEHTR
TERQVLEHIRQSPFLVTLHYAFQTETKLHLILDYINGGELFTHLSQRERFTEHEVQIYVGEI
VLALEHLHKLGIIYRDIKLENILLDSNGHVVLTDFGLSKEFVADETERAYSFCGTIEYMAP
DIVRGGDSGHDKAVDWWSLGVLMYELLTGASPFTVDGEKNSQAEISRRILKSEPPYPQE
MSALAKDLIQRLLMKDPKKRLGCGPRDADEIKEHLFFQKINWDDLAAKKVPAPFKPVIR
DELDVSNFAEEFTEMDPTYSPAALPQSSEKLFQGYSFVAPSILFKRNAAVIDPLQFHMGV
ERPGVTNVARSAMMKDSPFYQHYDLDLKDKPLGEGSFSICRKCVHKKSNQAFAVKIISK
RMEANTQKEITALKLCEGHPNIVKLHEVFHDQLHTFLVMELLNGGELFERIKKKKHFSE
TEASYIMRKLVSAVSHMHDVGVVHRDLKPENLLFTDENDNLEIKIIDFGFARLKPPDNQP
LKTPCFTLHYAAPELLNQNGYDESCDLWSLGVILYTMLSGQVPFQSHDRSLTCTSAVEI
MKKIKKGDFSFEGEAWKNVSQEAKDLIQGLLTVDPNKRLKMSGLRYNEWLQDGSQLSS
NPLMTPDILGSSGAAVHTCVKATFHAFNKYKREGFCLQNVDKAPLAKRRKMKKTSTST
ETRSSSSESSHSSSSHSHGKTTPTKTLQPSNPADSNNPETLFQFSDSVAGSDYKDDDDK

dCas9-dMSK1: amino acid sequence; 1 X “Flag” Epitope, Nuclear Localization Sequence,
Streptococcus pyogenes Cas9 (D10A, H840A), human MSK1(42-802) amino acid sequence
MDKKYSIGLAIGTNSVGWAVITDEYKVPSKKFKVLGNTDRHSIKKNLIGALLFDSGETA
EATRLKRTARRRYTRRKNRICYLQEIFSNEMAKVDDSFFHRLEESFLVEEDKKHERHPIF
GNIVDEVAYHEKYPTIYHLRKKLVDSTDKADLRLIYLALAHMIKFRGHFLIEGDLNPDNS
DVDKLFIQLVQTYNQLFEENPINASGVDAKAILSARLSKSRRLENLIAQLPGEKKNGLFG
NLIALSLGLTPNFKSNFDLAEDAKLQLSKDTYDDDLDNLLAQIGDQYADLFLAAKNLSD
AILLSDILRVNTEITKAPLSASMIKRYDEHHQDLTLLKALVRQQLPEKYKEIFFDQSKNGY
AGYIDGGASQEEFYKFIKPILEKMDGTEELLVKLNREDLLRKQRTFDNGSIPHQIHLGEL
HAILRRQEDFYPFLKDNREKIEKILTFRIPYYVGPLARGNSRFAWMTRKSEETITPWNFEE
VVDKGASAQSFIERMTNFDKNLPNEKVLPKHSLLYEYFTVYNELTKVKYVTEGMRKPA
FLSGEQKKAIVDLLFKTNRKVTVKQLKEDYFKKIECFDSVEISGVEDRFNASLGTYHDLL
KIIKDKDFLDNEENEDILEDIVLTLTLFEDREMIEERLKTYAHLFDDKVMKQLKRRRYTG
WGRLSRKLINGIRDKQSGKTILDFLKSDGFANRNFMQLIHDDSLTFKEDIQKAQVSGQG
DSLHEHIANLAGSPAIKKGILQTVKVVDELVKVMGRHKPENIVIEMARENQTTQKGQKN
SRERMKRIEEGIKELGSQILKEHPVENTQLQNEKLYLYYLQNGRDMYVDQELDINRLSD
YDVDAIVPQSFLKDDSIDNKVLTRSDKNRGKSDNVPSEEVVKKMKNYWRQLLNAKLIT
QRKFDNLTKAERGGLSELDKAGFIKRQLVETRQITKHVAQILDSRMNTKYDENDKLIRE
VKVITLKSKLVSDFRKDFQFYKVREINNYHHAHDAYLNAVVGTALIKKYPKLESEFVYG
DYKVYDVRKMIAKSEQEIGKATAKYFFYSNIMNFFKTEITLANGEIRKRPLIETNGETGEI
VWDKGRDFATVRKVLSMPQVNIVKKTEVQTGGFSKESILPKRNSDKLIARKKDWDPKK
YGGFDSPTVAYSVLVVAKVEKGKSKKLKSVKELLGITIMERSSFEKNPIDFLEAKGYKE
VKKDLIIKLPKYSLFELENGRKRMLASAGELQKGNELALPSKYVNFLYLASHYEKLKGS

PEDNEQKQLFVEQHKHYLDEIIEQISEFSKRVILADANLDKVLSAYNKHRDKPIREQAENI
IHLFTLTNLGAPAAFKYFDTTIDRKRYTSTKEVLDATLIHQSITGLYETRIDLSQLGGDKR
PAATKKAGQAKKKKGSEKVGIENFELLKVLGTGAYGKVFLVRKISGHDTGKLYAMKV
LKKATIVQKAKTTEHTRTERQVLEHIRQSPFLVTLHYAFQTETKLHLILDYINGGELFTHL
SQRERFTEHEVQIYVGEIVLALEHLHKLGIIYRDIKLENILLDSNGHVVLTDFGLSKEFVA
DETERAYSFCGTIEYMAPDIVRGGDSGHDKAVDWWSLGVLMYELLTGASPFTVDGEKN
SQAEISRRILKSEPPYPQEMSALAKDLIQRLLMKDPKKRLGCGPRDADEIKEHLFFQKIN
WDDLAAKKVPAPFKPVIRDELDVSNFAEEFTEMDPTYSPAALPQSSEKLFQGYSFVAPSI
LFKRNAAVIDPLQFHMGVERPGVTNVARSAMMKDSPFYQHYDLDLKDKPLGEGSFSIC
RKCVHKKSNQAFAVKIISKRMEANTQKEITALKLCEGHPNIVKLHEVFHDQLHTFLVME
LLNGGELFERIKKKKHFSETEASYIMRKLVSAVSHMHDVGVVHRDLKPENLLFTDENDN
LEIKIIDFGFARLKPPDNQPLKTPCFTLHYAAPELLNQNGYDESCDLWSLGVILYTMLSG
QVPFQSHDRSLTCTSAVEIMKKIKKGDFSFEGEAWKNVSQEAKDLIQGLLTVDPNKRLK
MSGLRYNEWLQDGSQLSSNPLMTPDILGSSGAAVHTCVKATFHAFNKYKREGFCLQNV
DKAPLAKRRKMKKTSTSTETRSSSSESSHSSSSHSHGKTTPTKTLQPSNPADSNNPETLFQ
FSDSVAGSDYKDDDDK

dCas9-ddMSK1: amino acid sequence; 1 X “Flag” Epitope, Nuclear Localization Sequence, Streptococcus pyogenes Cas9 (D10A, H840A), human MSK1(42-802, **D195A/D565A**) amino acid sequence

MDKKYSIGLAIGTNSVGWAVITDEYKVPSKKFKVLGNTDRHSIKKNLIGALLFDSGETA
EATRLKRTARRRYTRRKNRICYLQEIFSNEMAKVDDSFFHRLEESFLVEEDKKHERHPIF
GNIVDEVAYHEKYPTIYHLRKKLVDSTDKADLRLIYLALAHMIKFRGHFLIEGDLNPDNS
DVDKLFIQLVQTYNQLFEENPINASGVDAKAILSARLSKSRRLENLIAQLPGEKKNGLFG
NLIALSLGLTPNFKSNFDLAEDAKLQLSKDTYDDDLDNLLAQIGDQYADLFLAAKNLSD
AILLSDILRVNTEITKAPLSASMIKRYDEHHQDLTLLKALVRQQLPEKYKEIFFDQSKNGY
AGYIDGGASQEEFYKFIKPILEKMDGTEELLVKLNREDLLRKQRTFDNGSIPHQIHLGEL
HAILRRQEDFYPFLKDNREKIEKILTFRIPYYVGPLARGNSRFAWMTRKSEETITPWNFEE
VVDKGASAQSFIERMTNFDKNLPNEKVLPKHSLLYEYFTVYNELTKVKYVTEGMRKPA
FLSGEQKKAIVDLLFKTNRKVTVKQLKEDYFKKIECFDSVEISGVEDRFNASLGTYHDLL
KIIKDKDFLDNEENEDILEDIVLTLTLFEDREMIEERLKTYAHLFDDKVMKQLKRRRYTG
WGRLSRKLINGIRDKQSGKTILDFLKSDGFANRNFMQLIHDDSLTFKEDIQKAQVSGQG
DSLHEHIANLAGSPAIKKGILQTVKVVDELVKVMGRHKPENIVIEMARENQTTQKGQKN
SRERMKRIEEGIKELGSQILKEHPVENTQLQNEKLYLYYLQNGRDMYVDQELDINRLSD
YDVDAIVPQSFLKDDSIDNKVLTRSDKNRGKSDNVPSEEVVKKMKNYWRQLLNAKLIT
QRKFDNLTKAERGGLSELDKAGFIKRQLVETRQITKHVAQILDSRMNTKYDENDKLIRE
VKVITLKSKLVSDFRKDFQFYKVREINNYHHAHDAYLNAVVGTALIKKYPKLESEFVYG
DYKVYDVRKMIAKSEQEIGKATAKYFFYSNIMNFFKTEITLANGEIRKRPLIETNGETGEI
VWDKGRDFATVRKVLSMPQVNIVKKTEVQTGGFSKESILPKRNSDKLIARKKDWDPKK
YGGFDSPTVAYSVLVVAKVEKGKSKKLKSVKELLGITIMERSSFEKNPIDFLEAKGYKE
VKKDLIIKLPKYSLFELENGRKRMLASAGELQKGNELALPSKYVNFLYLASHYEKLKGS
PEDNEQKQLFVEQHKHYLDEIIEQISEFSKRVILADANLDKVLSAYNKHRDKPIREQAENI
IHLFTLTNLGAPAAFKYFDTTIDRKRYTSTKEVLDATLIHQSITGLYETRIDLSQLGGDKR
PAATKKAGQAKKKKGSEKVGIENFELLKVLGTGAYGKVFLVRKISGHDTGKLYAMKV
LKKATIVQKAKTTEHTRTERQVLEHIRQSPFLVTLHYAFQTETKLHLILDYINGGELFTHL
SQRERFTEHEVQIYVGEIVLALEHLHKLGIIYRDIKLENILLDSNGHVVLT**A**FGLSKEFVA

DETERAYSFCGTIEYMAPDIVRGGDSGHDKAVDWWSLGVLMYELLTGASPFTVDGEKN
SQAEISRRILKSEPPYPQEMSALAKDLIQRLLMKDPKKRLGCGPRDADEIKEHLFFQKIN
WDDLAAKKVPAPFKPVIRDELDVSNFAEEFTEMDPTYSPAALPQSSEKLFQGYSFVAPSI
LFKRNAAVIDPLQFHMGVERPGVTNVARSAMMKDSPFYQHYDLDLKDKPLGEGSFSIC
RKCVHKKSNQAFAVKIISKRMEANTQKEITALKLCEGHPNIVKLHEVFHDQLHTFLVME
LLNGGELFERIKKKKHFSETEASYIMRKLVSAVSHMHDVGVVHRDLKPENLLFTDENDN
LEIKII**A**FGFARLKPPDNQPLKTPCFTLHYAAPELLNQNGYDESCDLWSLGVILYTMLSG
QVPFQSHDRSLTCTSAVEIMKKIKKGDFSFEGEAWKNVSQEAKDLIQGLLTVDPNKRLK
MSGLRYNEWLQDGSQLSSNPLMTPDILGSSGAAVHTCVKATFHAFNKYKREGFCLQNV
DKAPLAKRRKMKKTSTSTETRSSSSESSHSSSSHSHGKTTPTKTLQPSNPADSNNPETLFQ
FSDSVAGSDYKDDDDK

dCas9-Aurora B, amino acid sequence; 1 X “Flag” Epitope, Nuclear Localization Sequence, Streptococcus pyogenes Cas9 (D10A, H840A), human Aurora B amino acid sequence
MDKKYSIGLAIGTNSVGWAVITDEYKVPSKKFKVLGNTDRHSIKKNLIGALLFDSGETA
EATRLKRTARRRYTRRKNRICYLQEIFSNEMAKVDDSFFHRLEESFLVEEDKKHERHPIF
GNIVDEVAYHEKYPTIYHLRKKLVDSTDKADLRLIYLALAHMIKFRGHFLIEGDLNPDNS
DVDKLFIQLVQTYNQLFEENPINASGVDAKAILSARLSKSRRLENLIAQLPGEKKNGLFG
NLIALSLGLTPNFKSNFDLAEDAKLQLSKDTYDDDLDNLLAQIGDQYADLFLAAKNLSD
AILLSDILRVNTEITKAPLSASMIKRYDEHHQDLTLLKALVRQQLPEKYKEIFFDQSKNGY
AGYIDGGASQEEFYKFIKPILEKMDGTEELLVKLNREDLLRKQRTFDNGSIPHQIHLGEL
HAILRRQEDFYPFLKDNREKIEKILTFRIPYYVGPLARGNSRFAWMTRKSEETITPWNFEE
VVDKGASAQSFIERMTNFDKNLPNEKVLPKHSLLYEYFTVYNELTKVKYVTEGMRKPA
FLSGEQKKAIVDLLFKTNRKVTVKQLKEDYFKKIECFDSVEISGVEDRFNASLGTYHDLL
KIIKDKDFLDNEENEDILEDIVLTLTLFEDREMIEERLKTYAHLFDDKVMKQLKRRRYTG
WGRLSRKLINGIRDKQSGKTILDFLKSDGFANRNFMQLIHDDSLTFKEDIQKAQVSGQG
DSLHEHIANLAGSPAIKKGILQTVKVVDELVKVMGRHKPENIVIEMARENQTTQKGQKN
SRERMKRIEEGIKELGSQILKEHPVENTQLQNEKLYLYYLQNGRDMYVDQELDINRLSD
YDVDAIVPQSFLKDDSIDNKVLTRSDKNRGKSDNVPSEEVVKKMKNYWRQLLNAKLIT
QRKFDNLTKAERGGLSELDKAGFIKRQLVETRQITKHVAQILDSRMNTKYDENDKLIRE
VKVITLKSKLVSDFRKDFQFYKVREINNYHHAHDAYLNAVVGTALIKKYPKLESEFVYG
DYKVYDVRKMIAKSEQEIGKATAKYFFYSNIMNFFKTEITLANGEIRKRPLIETNGETGEI
VWDKGRDFATVRKVLSMPQVNIVKKTEVQTGGFSKESILPKRNSDKLIARKKDWDPKK
YGGFDSPTVAYSVLVVAKVEKGKSKKLKSVKELLGITIMERSSFEKNPIDFLEAKGYKE
VKKDLIIKLPKYSLFELENGRKRMLASAGELQKGNELALPSKYVNFLYLASHYEKLKGS
PEDNEQKQLFVEQHKHYLDEIIEQISEFSKRVILADANLDKVLSAYNKHRDKPIREQAENI
IHLFTLTNLGAPAAFKYFDTTIDRKRYTSTKEVLDATLIHQSITGLYETRIDLSQLGGDKR
PAATKKAGQAKKKKGSMAQKENSYPWPYGRQTAPSGLSTLPQRVLRKEPVTPSALVL
MSRSNVQPTAAPGQKVMENSSGTPDILTRHFTIDDFEIGRPLGKGKFGNVYLAREKKSH
FIVALKVLFKSQIEKEGVEHQLRREIEIQAHLHHPNILRLYNYFYDRRRIYLILEYAPRGEL
YKELQKSCTFDEQRTATIMEELADALMYCHGKKVIHRDIKPENLLLGLKGELKIADFGW
SVHAPSLRRKTMCGTLDYLPPEMIEGRMHNEKVDLWCIGVLCYELLVGNPPFESASHNE
TYRRIVKVDLKFPASVPMGAQDLISKLLRHNPSERLPLAQVSAHPWVRANSRRVLPPSA
LQSVAGSDYKDDDDK

dCas9-Aurora B^{K106R}, amino acid sequence; 1 X “Flag” Epitope, Nuclear Localization Sequence, Streptococcus pyogenes Cas9 (D10A, H840A), human Aurora B (**K106R**) amino acid sequence

MDKKYSIGLAIGTNSVGWAVITDEYKVPSKKFKVLGNTDRHSIKKNLIGALLFDSGETAEATRLKRTARRRYTRRKNRICYLQEIFSNEMAKVDDSFFHRLEESFLVEEDKKHERHPIFGNIVDEVAYHEKYPTIYHLRKKLVDSTDKADLRLIYLALAHMIKFRGHFLIEGDLNPDNSDVDKLFIQLVQTYNQLFEENPINASGVDAKAILSARLSKSRRLENLIAQLPGEKKNGLFGNLIALSLGLTPNFKSNFDLAEDAKLQLSKDTYDDDLDNLLAQIGDQYADLFLAAKNLSDAILLSDILRVNTEITKAPLSASMIKRYDEHHQDLTLLKALVRQQLPEKYKEIFFDQSKNGYAGYIDGGASQEEFYKFIKPILEKMDGTEELLVKLNREDLLRKQRTFDNGSIPHQIHLGELHAILRRQEDFYPFLKDNREKIEKILTFRIPYYVGPLARGNSRFAWMTRKSEETITPWNFEEVVDKGASAQSFIERMTNFDKNLPNEKVLPKHSLLYEYFTVYNELTKVKYVTEGMRKPAFLSGEQKKAIVDLLFKTNRKVTVKQLKEDYFKKIECFDSVEISGVEDRFNASLGTYHDLLKIIKDKDFLDNEENEDILEDIVLTLTLFEDREMIEERLKTYAHLFDDKVMKQLKRRRYTGWGRLSRKLINGIRDKQSGKTILDFLKSDGFANRNFMQLIHDDSLTFKEDIQKAQVSGQGDSLHEHIANLAGSPAIKKGILQTVKVVDELVKVMGRHKPENIVIEMARENQTTQKGQKNSRERMKRIEEGIKELGSQILKEHPVENTQLQNEKLYLYYLQNGRDMYVDQELDINRLSDYDVDAIVPQSFLKDDSIDNKVLTRSDKNRGKSDNVPSEEVVKKMKNYWRQLLNAKLITQRKFDNLTKAERGGLSELDKAGFIKRQLVETRQITKHVAQILDSRMNTKYDENDKLIREVKVITLKSKLVSDFRKDFQFYKVREINNYHHAHDAYLNAVVGTALIKKYPKLESEFVYGDYKVYDVRKMIAKSEQEIGKATAKYFFYSNIMNFFKTEITLANGEIRKRPLIETNGETGEIVWDKGRDFATVRKVLSMPQVNIVKKTEVQTGGFSKESILPKRNSDKLIARKKDWDPKKYGGFDSPTVAYSVLVVAKVEKGKSKKLKSVKELLGITIMERSSFEKNPIDFLEAKGYKEVKKDLIIKLPKYSLFELENGRKRMLASAGELQKGNELALPSKYVNFLYLASHYEKLKGSPEDNEQKQLFVEQHKHYLDEIIEQISEFSKRVILADANLDKVLSAYNKHRDKPIREQAENIIHLFTLTNLGAPAAFKYFDTTIDRKRYTSTKEVLDATLIHQSITGLYETRIDLSQLGGDKRPAATKKAGQAKKKKGSMAQKENSYPWPYGRQTAPSGLSTLPQRVLRKEPVTPSALVLMSRSNVQPTAAPGQKVMENSSGTPDILTRHFTIDDFEIGRPLGKGKFGNVYLAREKKSHFIVAL**R**VLFKSQIEKEGVEHQLRREIEIQAHLHHPNILRLYNYFYDRRRIYLILEYAPRGELYKELQKSCTFDEQRTATIMEELADALMYCHGKKVIHRDIKPENLLLGLKGELKIADFGWSVHAPSLRRKTMCGTLDYLPPEMIEGRMHNEKVDLWCIGVLCYELLVGNPPFESASHNETYRRIVKVDLKFPASVPMGAQDLISKLLRHNPSERLPLAQVSAHPWVRANSRRVLPPSALQSVAGSDYKDDDDK
