# Supplementary material for: Programmable human histone phosphorylation and gene activation using a CRISPR/Cas9-based chromatin kinase
Source: Nat Commun. 2021 Feb 9;12:896. doi: 10.1038/s41467-021-21188-2 (PMC7873277; doi:10.1038/s41467-021-21188-2)
Supplement: Supplementary file 3 — Reporting Summary [file 41467_2021_21188_MOESM3_ESM.pdf]

nature research

Corresponding author(s): Isaac Hilton

Last updated by author(s): Jan 6, 2021

Reporting Summary

Nature Research wishes to improve the reproducibility of the work that we publish. This form provides structure for consistency and transparency in reporting. For further information on Nature Research policies, see our Editorial Policies and the Editorial Policy Checklist.

Statistics

For all statistical analyses, confirm that the following items are present in the figure legend, table legend, main text, or Methods section.

n/a	Confirmed	
[ ]	[x]	The exact sample size (*n*) for each experimental group/condition, given as a discrete number and unit of measurement
[ ]	[x]	A statement on whether measurements were taken from distinct samples or whether the same sample was measured repeatedly
[ ]	[x]	The statistical test(s) used AND whether they are one- or two-sided<br>*Only common tests should be described solely by name; describe more complex techniques in the Methods section.*
[x]	[ ]	A description of all covariates tested
[ ]	[x]	A description of any assumptions or corrections, such as tests of normality and adjustment for multiple comparisons
[ ]	[x]	A full description of the statistical parameters including central tendency (e.g. means) or other basic estimates (e.g. regression coefficient) AND variation (e.g. standard deviation) or associated estimates of uncertainty (e.g. confidence intervals)
[ ]	[x]	For null hypothesis testing, the test statistic (e.g. *F*, *t*, *r*) with confidence intervals, effect sizes, degrees of freedom and *P* value noted<br>*Give P values as exact values whenever suitable.*
[x]	[ ]	For Bayesian analysis, information on the choice of priors and Markov chain Monte Carlo settings
[x]	[ ]	For hierarchical and complex designs, identification of the appropriate level for tests and full reporting of outcomes
[x]	[ ]	Estimates of effect sizes (e.g. Cohen's *d*, Pearson's *r*), indicating how they were calculated

Our web collection on statistics for biologists contains articles on many of the points above.

Software and code

Policy information about availability of computer code

Data collection	Data was collected using commercially available platforms from Illumina.
Data analysis	HISAT2 version 2.1.0. FeatureCounts (v2.0.1), Bowtie2 (v2.3.4.2), SAMtools (v1.9), MACS2 (v2.1.2.1), BEDTools (v2.27.1), MAGeCK-VISPR (v0.5.4), R (v3.6.1), R studio (v1.2.13), DESeq2 (v3.11) and ggplot2 (v3.3.2).

For manuscripts utilizing custom algorithms or software that are central to the research but not yet described in published literature, software must be made available to editors and reviewers. We strongly encourage code deposition in a community repository (e.g. GitHub). See the Nature Research guidelines for submitting code & software for further information.

Data

Policy information about availability of data

All manuscripts must include a data availability statement. This statement should provide the following information, where applicable:
- Accession codes, unique identifiers, or web links for publicly available datasets
- A list of figures that have associated raw data
- A description of any restrictions on data availability

RNA sequencing data, ChIP-seq and CRISPR screening data supporting this study is deposited as GSE156381.

Field-specific reporting

Life sciences study design

All studies must disclose on these points even when the disclosure is negative.

Sample size	Generally, sample sizes were chosen to meet or exceed the standards of reproducibility demonstrated in similar published studies.
Data exclusions	No data were excluded from the manuscript.
Replication	Experiments were replicated at least three times on different days using the reported methods. All results were reliably replicated.
Randomization	This is not relevant as commercial cell lines were used for our experiments.
Blinding	Blinding was not possible as experimental conditions were evident to experimenters.

Reporting for specific materials, systems and methods

We require information from authors about some types of materials, experimental systems and methods used in many studies. Here, indicate whether each material, system or method listed is relevant to your study. If you are not sure if a list item applies to your research, read the appropriate section before selecting a response.

Materials & experimental systems

n/a	Involved in the study	
☐	☒	Antibodies
☐	☒	Eukaryotic cell lines
☒	☐	Palaeontology and archaeology
☒	☐	Animals and other organisms
☒	☐	Human research participants
☒	☐	Clinical data
☒	☐	Dual use research of concern

Methods

n/a	Involved in the study	
☐	☒	ChIP-seq
☒	☐	Flow cytometry
☒	☐	MRI-based neuroimaging

Antibodies

Antibodies used	α-FLAG (Mouse,Sigma-Aldrich, #F1804, 1:1000 for western blotting and 1:100 for ChIP), α-mouse IgG HRP (Rabbit, Sigma-Aldrich, #A6154,1:3000), α-MSK1 (Rabbit, Abcam, #ab99412,1:1000 for western blotting and 1:100 for ChIP), α-MSK1S376ph (Rabbit, Abcam, #ab32190,1:1000), α-MSK1S212ph (Rabbit, Abcam, #ab79499,1:1000), α-rabbit IgG HRP(Goat, Abcam, #ab6721,1:3000), hFAB™ Rhodamine Anti-Tubulin Primary Antibody (Bio-Rad, #12004166,1:3000); α-H3S10ph (Rabbit, Abcam, #5176,1:1000), α-H3S10ph (Abcam, #17246, 1:100),α-H3S28ph (Rabbit, Abcam, #ab32388,1:1000 for western blotting and 1:00 for ChIP), α-H3 (Rabbit, Abcam, #1791,1:1000), Rabbit IgG (Abcam, ab171870,1:100), Mouse IgG (Abcam, #18413,1:100), α-H3K4me3 (Abcam, #ab8580.1:100)
Validation	α-FLAG (Sigma-Aldrich, #F1804) was verified for western blotting (Seymour M et al, G3 (Bethesda). 2016, 6(7): 1879–1889) and for ChIP grade (Hojo et al., Developmental Cell. 2016, 37:238–253.)<br>α-mouse IgG HRP (Sigma-Aldrich, #A6154) was verified for western blotting (Nurwakagar P et al, J Mol Endocrinol. 2007, 38 (1-2):259-275). α-MSK1 (Abcam, #ab99412), α-MSK1S376ph (Abcam, #ab32190) and α-MSK1S212ph (Abcam, #ab79499) have been verified for western bloting by manufacturer (see product website). α-rabbit IgG HRP(Abcam, #ab6721) has been validated for western blot (Guo Z et al, Int J Mol Med, 2020, 45:141-150). α-H3 (Abcam, #1791) has been verified for western blot (Ma X et al. Proc Natl Acad Sci U S A. 2020, 117:761-770). hFAB™ Rhodamine Anti-Tubulin Primary Antibody (Bio-Rad, #12004166) have been validated for western blot (see product website). α-H3S10ph (Abcam, #5176), has been validated for western blot (see product website). α-H3S28ph (Abcam, #ab32388) has been validated for western blot (Alexandrova EM et al. Nature. 2015, 523:352-356) and ChIP-grade (see product website). α-H3S10ph (Abcam, #17246), has been validated for ChIP grade (see product website).<br>α-Rabbit IgG (Abcam, ab171870) has been validated for ChIP grade (Wang Y et al. Alzheimers Res Ther. 2020, 12:29).<br>α-Mouse IgG (Abcam, #18413) has been validated for ChIP grade (Lian SL et al. Immun Inflamm Dis. 2018, 6:58-71).<br>α-H3K4me3 (Abcam, #ab8580) has been validated for ChIP grade (Han M et al. EMBO Mol Med . 2020, 12:e10924).

Eukaryotic cell lines

Policy information about cell lines

Cell line source(s)	HEK293T, A375 and A549 cells were purchased new from the ATCC.
Authentication	None of the cell lines were authenticated.
Mycoplasma contamination	Cell lines were not tested for Mycoplasma contamination.

Commonly misidentified lines (See ICLAC register)	No commonly misidentified cell lines were used.

ChIP-seq

Data deposition

☒ Confirm that both raw and final processed data have been deposited in a public database such as GEO.

☒ Confirm that you have deposited or provided access to graph files (e.g. BED files) for the called peaks.

Data access links<br>*May remain private before publication.*	https://www.ncbi.nlm.nih.gov/geo/query/acc.cgi?acc=GSE156381
Files in database submission	FLAG1_S3_L001_R1_001.fastq.gz<br>FLAG2_S4_L001_R1_001.fastq.gz<br>FLAG3_S5_L001_R1_001.fastq.gz<br>FLAG4_S6_L001_R1_001.fastq.gz<br>S28Ph1_S7_L001_R1_001.fastq.gz<br>S28Ph2_S8_L001_R1_001.fastq.gz<br>S28Ph1_S74_L008_R1_001.fastq.gz<br>S28Ph2_S75_L008_R1_001.fastq.gz<br>S28Ph3_S9_L001_R1_001.fastq.gz<br>S28Ph4_S10_L001_R1_001.fastq.gz<br>S28Ph3_S76_L008_R1_001.fastq.gz<br>S28Ph4_S77_L008_R1_001.fastq.gz<br>Input12_S1_L001_R1_001.fastq.gz<br>Input34_S2_L001_R1_001.fastq.gz<br>FLAG_OCT4_targeted_re1.bdg<br>FLAG_OCT4_targeted_re2.bdg<br>FLAG_non_targeting_re1.bdg<br>FLAG_non_targeting_re2.bdg<br>S28ph_OCT4_targeted_pooled.bdg<br>S28ph_non_targeting_pooled.bdg
Genome browser session (e.g. UCSC)	No longer applicable.

Methodology

Replicates	2
Sequencing depth	Single-end reads of 50 bp<br>Sample name #of Reads #of Unique Reads<br>FLAG1_S3_L001_R1_001.fastq.gz, 25869624,20709682<br>FLAG2_S4_L001_R1_001.fastq.gz, 27554785,21734732<br>FLAG3_S5_L001_R1_001.fastq.gz, 26828692,22367182<br>FLAG4_S6_L001_R1_001.fastq.gz, 26933661, 22831458<br>Input12_S1_L001_R1_001.fastq.gz,22564649, 19273403<br>Input34_S2_L001_R1_001.fastq.gz, 23041464, 19761374<br>ChIP-seq sample pool of (S28Ph1_S7_L001_R1_001.fastq.gz, S28Ph2_S8_L001_R1_001.fastq.gz, S28Ph1_S74_L008_R1_001.fastq.gz,S28Ph2_S75_L008_R1_001.fastq.gz) total reads: 108699345. Unique reads: 101598139<br>ChIP-seq sample pool of (S28Ph3_S9_L001_R1_001.fastq.gz, S28Ph4_S10_L001_R1_001.fastq.gz, S28Ph3_S76_L008_R1_001.fastq.gz, S28Ph4_S77_L008_R1_001.fastq.gz) total reads: 134197339. Unique reads: 128336394
Antibodies	α-FLAG (Sigma-Aldrich, #F1804) and α-H3S28ph (Abcam, #ab32388) have been validated for ChIP grade by manufacturer and peer reviewed publications.

Peak calling parameters	Software used MACS2 version v2.1.2.1<br>Genome version hg38 from UCSC<br>treatment :-t OCT4 targeting or non-targeting<br>control :-c OCT4 targeting or non-targeting_Input input file format: -f bam<br>effective genome size: -g hg<br>q-value cut-off:--broad-cutoff 0.01<br>command :macs2 callpeak
Data quality	reads were aligned to the hg38 reference genome by Bowtie2 (v2.3.4.2) and removed PCR duplicates using the rmdup tool from SAMtools (v1.9). Reads from each duplicate for each condition were combined, and peaks were called using MACS2 (v2.1.2.1). Resulting peaks from each condition with a q-value ≤ 0.01 were merged using the mergeBed tool from BEDTools (v2.27.1).<br>For FLAG ChIP-seq, 6279 peaks with FDR < 0.01.<br>For S28ph ChIP-seq, 349 peaks were detected with FDR < 0.01
Software	Alignment tools: Bowtie2 (v2.3.4.2)<br>Sorting tools: SAMtools (v1.9)<br>Peak calling tools: MACS2 (v2.1.2.1).
